# Heat shock protein 90 is a chaperone regulator of HIV-1 latency

**DOI:** 10.1371/journal.ppat.1012524

**Published:** 2025-04-01

**Authors:** Somaya Noorsaeed, Nawal AlBurtamani, Ahmed Rokan, Ariberto Fassati

**Affiliations:** 1 Department of Medical Laboratory Sciences, Faculty of Applied Medical Sciences, King Abdulaziz University, Jeddah, Saudi Arabia; 2 Division of Infection & Immunity and Institute of Immunity and Transplantation, University College London, London, United Kingdom; 3 Department of Medical Laboratory Sciences, College of Applied Medical Sciences, Prince Sattam Bin Abdulaziz University, Alkharj, Saudia Arabia; University of Iowa, UNITED STATES OF AMERICA

## Abstract

An estimated 32 million people live with HIV-1 globally. Combined antiretroviral therapy suppresses viral replication but therapy interruption results in viral rebound from a latent reservoir mainly found in memory CD4+ T cells. Treatment is therefore lifelong and not curative. Eradication of this viral reservoir requires hematopoietic stem cell transplantation from hemizygous or homozygous ΔCCR5 donors, which is not broadly applicable. Alternative cure strategies include the pharmacological reactivation of latently infected cells to promote their immune-mediated clearance, or the induction of deep latency. HIV-1 latency is multifactorial and linked to the activation status of the infected CD4+ T cell. Hence to perturb latency, multiple pathways need to be simultaneously targeted without affecting CD4+ T cell function. Hsp90 has been shown to regulate HIV-1 latency, although knowledge on the pathways is limited. Because Hsp90 promotes the proper folding of numerous cellular proteins required for HIV-1 gene expression, we hypothesized that Hsp90 might be a master regulator of latency. We tested this hypothesis using a polyclonal Jurkat cell model of latency and ex-vivo latently infected primary CD4+ T cells. We found that, in the Jurkat model, Hsp90 is required for HIV-1 reactivation mediated by the T-cell receptor, phorbol esters, TNF-α, inhibition of FOXO-1, and agonists of TLR-7 and TLR-8. In primary cells, Hsp90 regulates HIV-1 gene expression induced by stimulation of the T-cell receptor or in the presence of IL-7/IL-15 or a FOXO-1 inhibitor. Chemical inhibition of Hsp90 abrogated activation of the NF-kB, NFAT and AP-1 signal transduction pathways. Within the CD4+ T cell population, CDRA45+ CCR7+ “naïve” and CD45RA- CCR7- “effector memory” cells were most sensitive to Hsp90 inhibition, which did not perturb their phenotype or activation state. Our results indicate that Hsp90 is a master regulator of HIV-1 latency that can potentially be targeted in cure strategies.

## Author summary

HIV-1 affects around 32 million people globally. Current treatments, known as combined antiretroviral therapy, can suppress the virus but do not cure the infection and if the treatment stops, the virus comes back. This happens because the virus hides in a population of immune cells called memory CD4+ T cells. To truly cure HIV-1, some strategies involve complex and risky procedures like hematopoietic stem cell transplants, which are not widely applicable. Another approach is to reactivate the hidden virus in the cells, so the immune system can eliminate it, or to force the virus into an even deeper hiding state. HIV-1 latency, or its ability to hide in cells, is influenced by many factors and cells need to be activated to disrupt it. Hsp90 is a chaperone that regulates the function of numerous proteins important for HIV-1 latency and is known to play a role in maintaining this hidden state of the virus. We therefore wondered if Hsp90 acts like a master regulator of latency. Using lab-based models, we discovered that Hsp90 is crucial for the reactivation of HIV-1 through various pathways. By inhibiting Hsp90, the activation of key signalling pathways necessary for viral reactivation was blocked. Importantly, blocking Hsp90 did not harm the CD4+ T cells’ function or state. Hsp90 inhibitors, already tested in cancer treatments, could thus be a promising avenue for HIV-1 cure strategies, as they seem to hold the key to maintaining HIV-1 latency.

## Introduction

Untreated human immunodeficiency virus type 1 (HIV-1) infection causes a progressive loss of CD4+ T cells that eventually results in death of the infected individual due to profound immunodeficiency, opportunistic infections, neurocognitive deficits and cancer such as Kaposi sarcoma [1,2]. Currently, combined antiretroviral therapy (cART) extends the life expectancy of people living with HIV-1 (PLWH) who are virologically suppressed to levels almost equal to uninfected individuals [3]. Treatment with cART must be lifelong because its interruption results in rapid virological rebound from a latently infected cell reservoir, even if therapy started early post-infection [4]. The largest reservoir is constituted by memory CD4+ T cells in which HIV-1 has integrated and established a transcriptionally silent infection, although infected tissue resident macrophages and microglial cells in the brain also contribute to the reservoir [5,6]. The latent reservoir is long-lived due to homeostatic proliferation of latently infected memory CD4+ T cells, their clonal expansion and immune selection, and the ability of intact but silent HIV-1 proviruses to reactivate upon stimulation, restarting the replication cycle [5,7]. Thus, cART cannot cure HIV-1 infection. To compound this problem, during cART, many PLWH show persistent immune activation due to gut microbial translocation, co-infections and viral antigen expression from a proportion of both intact and defective HIV-1 proviruses [8]. Although cART has mostly eliminated AIDS-defining illnesses, persistent immune activation significantly increases the odds developing co-morbidities in PLWH [9].

A cure by eradicating HIV-1 infected cells has been achieved only by allogeneic hematopoietic stem cell transplantation using hemizygous or homozygous ΔCCR5 donors, but this procedure is not broadly applicable to PLWH [10]. However, approaches to achieve long-term remission after therapy interruption are being developed [11]. One approach combines latency reactivating agents (LRAs) to stimulate viral antigen expression followed by immune-mediated clearance of infected cells. Another approach aims at repressing HIV-1 reactivation and generating a state of deep, potentially irreversible latency [11]. For optimal results, these two strategies require a detailed understanding of how HIV-1 latency is regulated and maintained.

HIV-1 gene expression is driven by the enhancer and promoter regions in the viral long terminal repeats (LTRs). Initiation of HIV-1 transcription relies on the transcription factors NFAT, NF-kB, AP-1 (c-Fos/c-Jun) and the ras-responsive binding factor-2 (RBF-2), which bind to cognate cis-acting sequences in the LTR [12–14]. Notably, these transcription factors determine not just HIV-1 transcriptional initiation, but also many of the cellular response to T-cell receptor (TCR) stimulation, demonstrating the close link between T-cell and viral transcriptional regulation [15–17]. Upon transcriptional initiation, the viral protein Tat is produced and imported into the nucleus where it recruits the host positive transcription elongator factor-b (P-TEFb) on to the LTR [18,19]. P-TEFb, a heterodimer complex formed by cyclin T1 (CyT1) and the kinase subunit CDK9, boosts processivity of RNA polymerase (RNAPol) II by phosphorylating its C-terminal domain and triggering the release of negative regulators such as DSIF and NELF [18,19]. This is followed by the epigenetic reorganization of chromatin at the site of viral integration, leading to sustained viral gene expression [18,19].

HIV-1 latency is established at multiple levels, including the integration site in the host genome, the epigenetic configuration of chromatin at or near the site of integration, the activation state of the infected CD4+ T cells, which determines the nuclear availability of critical transcription factors, the abundance of P-TEFb, and the recruitment of transcriptional and post-transcriptional repressive factors [18,19].

Inhibiting HIV-1 gene expression should prevent HIV-1 reactivation from latency and reduce chronic inflammation in PLWH, yet there are no approved drugs that target this step of the viral life cycle, and only a few are at the development or pre-clinical stage. Examples include PKC, PI3K and MEK inhibitors, which have been reported to suppress HIV-1 latency reactivation *in vitro* by inhibiting TCR mediated stimulation of latently infected CD4+ T cells [13,20]. Aryl hydrocarbon receptor (AhR) agonists [21] and RORC2 antagonists have been reported by our group to block viral outgrowth after stimulation of patients’ latently infected CD4+ T cells [22]. One limitation of this approach is that HIV-1 reactivation from latency depends on several parallel signalling pathways [19] hence blocking one pathway at a time may not be sufficient to achieve measurable silencing of viral gene expression. In addition, the effect of these drugs on CD4+ T cell behaviour is poorly understood.

This limitation can be addressed by targeting a viral protein that is essential for HIV-1 gene expression. An analogue of cortistatin A (didehydro-cortistatin A or dCA) has been shown to suppress Tat activity both *in vitro* and in humanized mice, resulting in specific inhibition of HIV-1 reactivation, and fostering epigenetic modifications that maintain latency longer term [23–25]. A complementary strategy is to target master regulators that control multiple parallel pathways required for HIV-1 reactivation. Thus, there is a clear rationale to develop pharmacological interventions that repress HIV-1 reactivation from latency by targeting multiple pathways simultaneously, provided that these interventions do not perturb the overall T cell function.

We and others have demonstrated that heat shock protein 90 (Hsp90) is a host factor required for HIV-1 gene expression and reactivation from latency [13,26–33]. The chaperone seems to act mainly at the early stages of viral gene expression [26,27,32,34]. Hsp90 and its co-chaperone Cdc37 were found to promote the activation of the NF-kB pathway by ensuring the correct assembly and function of the IKK complex [13]. Hsp90 also co-localized with actively transcribing proviruses [13] and maintained the function of P-TEFb [32,34]. Notably, treatment of latently infected cells with selective Hsp90 inhibitors *in vitro* and in humanized mice repressed HIV-1 reactivation, and this effect was long-lasting [13,28,31,32,34]. Several selective Hsp90 inhibitors have been tested in phase II and III clinical trials to treat haematological malignancies and solid tumours [35]. The pharmacokinetics and pharmacodynamics of these drugs are well understood, and the drug concentration sufficient to inhibit HIV-1 reactivation is significantly lower than that one required for the anti-cancer effect [13,28,35,36], paving the way to their possible repurposing to inhibit HIV-1 reactivation in PLWH.

Hsp90 is a very conserved molecular chaperone that is responsible for folding, stabilizing, and activating a broad variety of client proteins. It operates as part of a large protein complex that includes co-chaperones, such as Hsp70, and other regulatory factors that control the activity and stability of client proteins. Hsp90 is known to facilitate many cellular processes, including protein homeostasis, signal transduction, and stress response [37]. Because Hsp90 promotes the correct folding and assembly of many host factors required for HIV-1 gene expression [13,27,28,31,34,38], we hypothesized that Hsp90 might be a master regulator of HIV-1 reactivation from latency. Here, we tested this hypothesis using a polyclonal Jurkat cell model of latency and ex-vivo latently infected primary CD4+ T cells, and we evaluated how inhibiting Hsp90 affects the activation and differentiation of CD4+ T cells.

## Results

### Establishment of a polyclonal Jurkat model of HIV-1 latency

**Fig 1:**
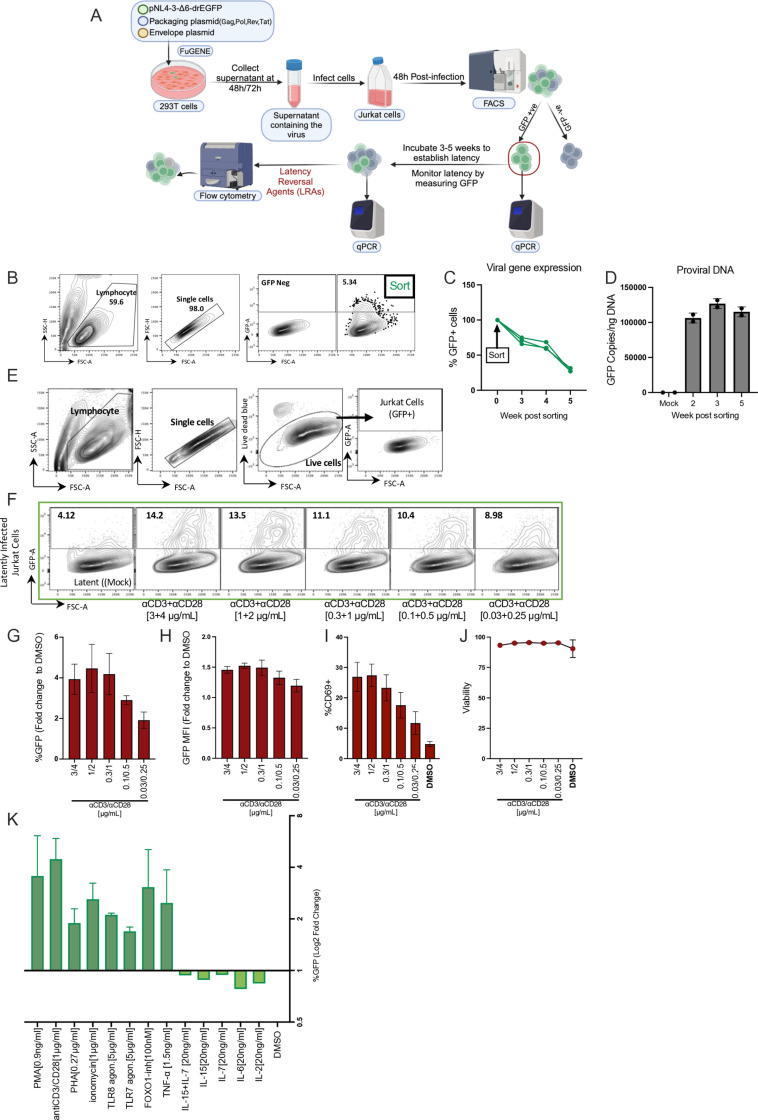
Establishment of a polyclonal Jurkat model of HIV-1 latency. (A) Schematic description of the generation of the latent Jurkat cell model. An HIV-1 reporter virus (pNL4-3-Δ6-drEGFP) encoding for destabilized GFP under the control of the HIV-1 promoter/enhancer (LTR) was used to differentiate between active and latently infected cells. pNL4-3-Δ6-drEGFP is ΔEnv and ΔNef and has six premature stop codons in gag, vif, vpr, and vpu. The virus only allows for one round of infection. The VSV-G pseudotyped HIV-1 vector was produced by FuGENE transfection into 293T cells. The produced virus was used to infect Jurkat cells, and infection was confirmed by flow cytometry. Forty-eight hours post-infection, GFP+ (infected) cells were sorted by Fluorescence-Activated Cell Sorting (FACS) and maintained until latent infection was established. (B) Flow cytometry gating strategy used for GFP+ cell sorting and monitoring of latency. (C) Flow cytometry analysis for GFP expression at different time points after sorting the infected cells. Three experiments are shown. (D) Quantification by qPCR of proviral DNA copies (using GFP primers) in mock-infected cells and sorted GFP+ cells at different weeks post-sorting. (E) Flow cytometry gating strategy used for detection of GFP+ cells after addition of different LRAs. Uninfected Jurkat cells were used to set the GFP+ gate. (F) Representative flow cytometer plots for anti-CD3/CD28 Ab titration. Latently infected Jurkat cells were activated with five different concentrations of anti-CD3/CD28 Ab or DMSO for 24 hours. The frequency of GFP+ T cells was quantified using flow cytometry; the gating strategy in E was used to detect GFP+ cells. (G) Bar plots showing the percentage of GFP+ cells (fold change to DMSO) after exposure to the indicated concentration of stimuli. (H) Bar graphs showing the GFP MFI fold change to DMSO. (I) Bar graphs showing the % CD69+ cells after anti-CD3/CD28 treatment. Bar plots show average values ± SD, n =6. (J) Viability was measured by flow cytometry using a blue live/dead stain. (K) Summary of latent viral reactivation in Jurkat cells; bar graphs show Log2 fold changes in the percentage of GFP+ cells over DMSO (negative control) for the specified concentration of each LRA. Schematic in (A) was created with BioRender.com.

To investigate if Hsp90 is a master regulator of HIV-1 latency, we first established a cell model of latency in which a variety of latency reversing agents (LRAs) and their dependence on Hsp90 could be easily tested. The J-Lat cell clones have been very useful to study the regulation of HIV-1 latency and many of the key findings that emerged using this model have been confirmed in primary CD4+ T cells and in cells from PLWH [39–42]. However, for the purpose of this study, the clonality of the J-Lat model, in which each clone has a single provirus integrated at a specific location, was a limitation. Because the site of integration affects HIV-1 gene expression [43–45], several J-Lat clones would need to be tested to approximate the polyclonal nature of the latent reservoir *in vivo*, making screenings complicated. To obviate this limitation, we established a polyclonal Jurkat cell model of latency (Fig 1A). Briefly, Jurkat cells were infected with a VSV-G-pseudotyped single cycle HIV-1 vector (pNL4.3Δ6-drGFP) [46] (kind gift of Dr. Robert Siliciano, Johns Hopkins University) that expresses a destabilized GFP whose transcription is driven by the viral LTR. This HIV-1 construct has two stop codons in gag-pol and so a separate gag-pol plasmid was provided during transfection of 293T cells to generate the viral supernatants [46]. Infected cells were sorted to select a pure population of GFP+ cells (Fig 1B), which were expanded for 5 weeks until latency (GFP- cells) was apparent (Fig 1C). Quantitative PCR to detect the GFP gene in the infected cells showed no loss of proviral DNA during passaging (Fig 1D), demonstrating actual viral latency.

We then surveyed 12 different LRAs that were previously shown to be active in latently infected primary cells, including cells from PLWH [16,40,46–48] (S1 Table). These LRAs are known to act through either distinct, or partially overlapping mechanisms (S1 Table) and were therefore suited broadly to probe the pathways for HIV-1 reactivation in our polyclonal latency model. Each LRA was tested at different concentrations to determine the dose-response profile and assess cell toxicity (S2 Table). Latently infected cells were exposed to the LRAs for 24-48h then analysed by flow cytometry to measure the percentage of GFP+ cells and the GFP mean fluorescent intensity (MFI) as surrogate markers of HIV-1 gene expression. CD69 was used as an early marker of CD4+ T cells activation [49]. Stimulation of the TCR by anti-CD3 and anti-CD28 antibodies showed a dose-dependent increase in both the percentage of GFP+ cells and their MFI relative to untreated cells (Fig 1E–1H). TCR stimulation up-regulated surface expression of CD69, as expected, and did not cause loss of cell viability (Fig 1I and 1J). Phorbol 12-myristate 13-acetate (PMA) and Phytohemagglutinin (PHA) also showed a good dose-response in terms of percentage of GFP+ cells, MFI and simultaneous up-regulation of CD69 (S1A and S1B Fig). Ionomycin induced HIV-1 reactivation and CD69 expression in a stepwise fashion above a certain concentration (S1C Fig).

Toll-like receptor 7 (TLR7) and TLR8 agonists have been reported to reactivate latent HIV-1 in CD4+ T cells by inducing an inflammatory response [48,50]. We therefore tested if TLR7 and TLR8 agonists promoted reactivation in our model. Treatment of the cells with TLR7 and TLR8 agonists caused a dose-dependent increase in both the percentage of GFP+ cells and MFI but did not significantly affect CD69 expression (S1D Fig). FOXO-1 contributes to the maintenance of HIV-1 latency by promoting a quiescent state in infected CD4+ T cells and inhibitors of FOXO-1 can reactivate the latent virus [51,52]. We therefore tested a well-characterized selective FOXO-1 inhibitor [51,52] in our cell model and confirmed previous reports by showing that the compound reactivated HIV-1 without affecting cell viability or CD69 expression (S1E Fig) [51,52]. This supports the notion that the HIV-1 and CD69 promoters share some but not all the regulatory pathways [46]. Treatment with TNF-α induced viral reactivation with no CD69 upregulation or loss of cell viability (S2A Fig) whereas cytokines IL-7, IL-15, IL-6 and IL-2 were inactive in our model (S2B–S2D Fig). The inactivity of IL-7 can be explained by low surface expression of the IL-7 receptor CD127 in Jurkat cells (S2E Fig). In summary, 9 known LRAs induced HIV-1 reactivation in our polyclonal model, demonstrating its suitability to test the role of Hsp90 (Fig 1K).

### Inhibition of Hsp90 broadly suppresses HIV-1 reactivation

**Fig 2:**
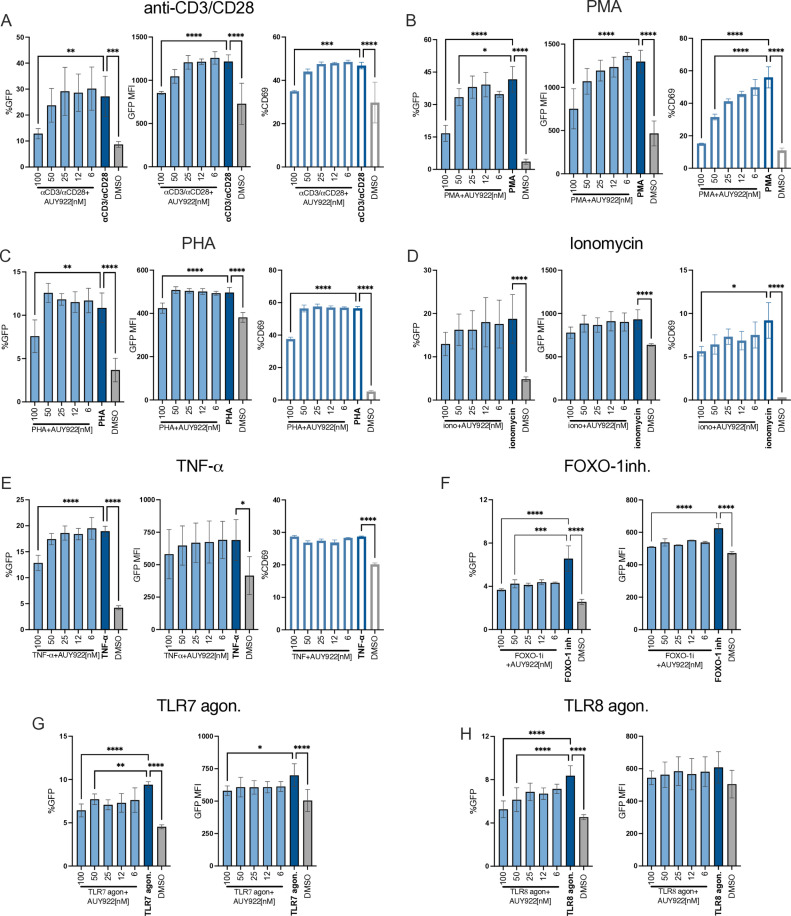
AUY922 represses HIV-1 reactivation in latently infected Jurkat cells. Latently infected Jurkat cells were stimulated with (A) anti-CD3/CD28 Abs [1μg/ml/2μg/ml], (B) PMA [0.9ng/ml], (C) PHA [0.27μg/ml], (D) ionomycin [1 μg/ml], (E) TNF-α [1.5 ng/ml], (F) FOXO-1 inh [200 nM], (G) TLR7 agonist [5μg ml] (H) TLR8 agonist [5μg/ml], or DMSO to induce HIV-1 reactivation. At the time of stimulation, cells were also treated with the indicated concentrations of AUY922 for 24h and analysed by flow cytometry. In (A-H), bar plots in the left panels show the average percentage ± SD (n=6) of GFP+ cells, middle panels show the average GFP MFI ± SD (n=6), and right panels show the average percentage ± SD (n=3) of CD69^+^ cells. Statistical significance was calculated using one-way ANOVA with Dunnett’s correction. *=p≤0.05; **=p≤0.01; ***=p≤0.001;****=p<0.0001.

To test if the LRAs shown in Fig 1K were dependent on Hsp90, we employed AUY922, a selective and well characterized Hsp90 inhibitor that binds to its N-terminal ATPase pocket and outcompetes ATP [53,54]. Latently infected cells were treated for 24 hours with a fixed concentration of each LRA in the presence of increasing concentrations of AUY922, or DMSO as control. The LRA concentration chosen had to be within the linear range of the dose-response curve in the absence of cell toxicity. AUY922 was added at the same time as the LRA, and we did not see a difference if AUY922 was added 30 minutes before the LRA, presumably because of the good cell permeability of the drug [55]. Treated cells were analysed by flow cytometry 24 hours after treatment except for the FOXO-1 inhibitor which was analysed after 48 hours. AUY922 inhibited HIV-1 reactivation mediated by TCR stimulation (Fig 2A), PMA (Fig 2B), PHA (Fig 2C), TNF-α(Fig 2E), the FOXO-1 inhibitor (Fig 2F) and the TLR7 and TLR8 agonists (Fig 2G and 2H). AUY922 did not appear to be significantly active against ionomycin-induced HIV-1 reactivation even if it antagonized ionomycin-induced upregulation of CD69 surface expression (Fig 2D). In general, AUY922 reduced the percentage of GFP+ cells more than the GFP MFI, suggesting that only a proportion of latently infected cells were responding to treatment. This uneven response was reported before in latently infected cells and is presumably related to the stochastic nature of transcription and the chromatin landscape surrounding the provirus [56,57]. In the future, this can be tested by using J-Lat clones in which the response to AUY922 may be correlated to the specific proviral integration site. No loss of cell viability was detected in cells treated with the LRAs and AUY922, except in samples treated with the TLR8 agonist and AUY922 at 100nM (S3 Fig).

**Fig 3:**
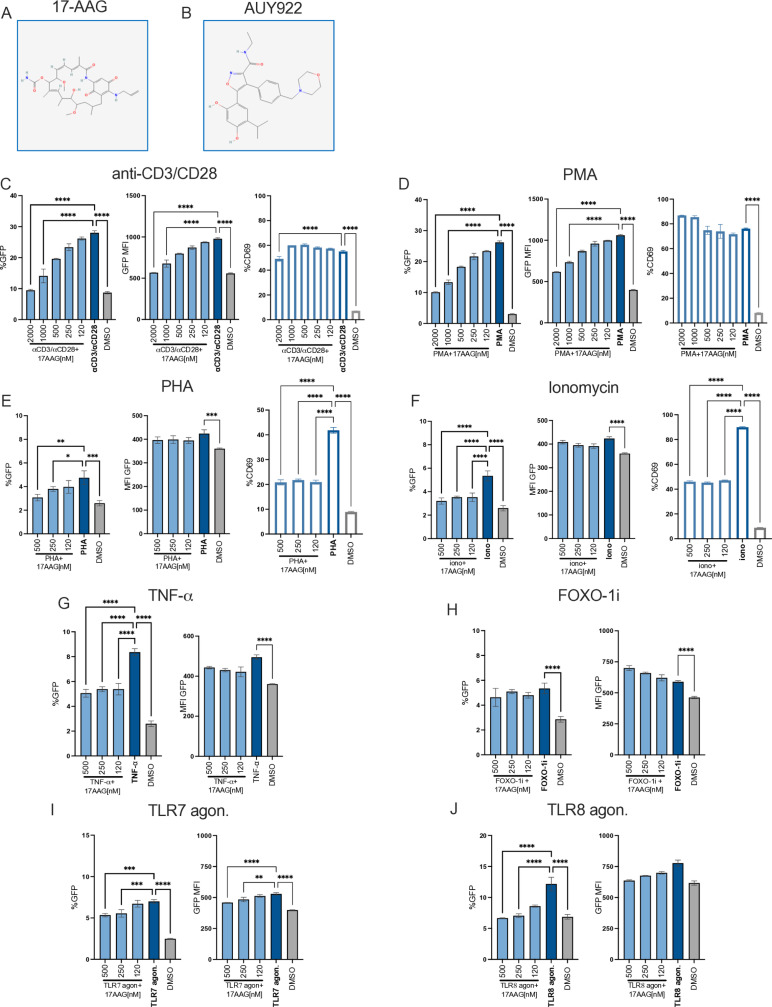
Inhibition of HIV-1 reactivation by 17-AAG in latently infected Jurkat cells. (A) Chemical structure of 17-AAG (PubChem ID: 6505803). (B) Chemical structure of AUY922 (PubChem ID:135539077). (C-F) Latently infected Jurkat cells were stimulated with (C) anti-CD3/CD28 Abs, (D) PMA, (E) PHA, (F) Ionomycin, (G) TNF-α, (H) FOXO-1 inhibitor, (I) TLR7 agonist or (J) TLR8 agonist or DMSO for 24 hours to induce HIV-1 reactivation. At the time of stimulation, cells were also treated with the indicated concentrations of 17-AAG for 24h and analysed by flow cytometry. In (C-F), left panels show the average percentage ± SD (n=3) of GFP+ cells, middle panels show the average GFP MFI ± SD (n=3) and right panels show the average percentage ± SD (n=3) of CD69^+^ cells. In (G-J) the left panels show the average percentage ± SD (n=3) of GFP+ cells and the right panels show the average GFP MFI ± SD (n=3). Statistical significance was calculated using one-way ANOVA with Dunnett’s correction. *=p≤0.05; **=p≤0.01; ***=p≤0.001; ****=p<0.0001.

To confirm that these effects were indeed dependent on Hsp90 inhibition, we employed the benzoquinone ansamycin 17-allylamino-17-demethoxygeldanamycin (17-AAG or tanespimycin), a well-characterised Hsp90 inhibitor that binds to the ATPase pocket of Hsp90 but has a different chemical structure from AUY922 (Fig 3A and 3B) [54,58]. The experimental design with 17-AAG was the same as with AUY22. A fixed concentration of LRAs was added to the cells for 24–48 hours then cells were analysed by flow cytometry to measure the percentage of GFP+ cells, GFP MFI, CD69 and cell viability. 17-AAG antagonised the viral reactivation induced by TCR stimulation (Fig 3C), PMA (Fig 3D), PHA (Fig 3E), ionomycin (Fig 3F), TNF-α (Fig 3G) and the TLR7 and TLR8 agonists (Fig 3I and 3J) with no loss of cell viability (S4 Fig). However, 17-AAG did not significantly abrogate the reactivation induced by the FOXO-1 inhibitor (Fig 3H). Taken together, the results demonstrate that Hsp90 broadly regulates HIV-1 reactivation, including that one triggered by TLR7 and TLR8 activation and inhibition of FOXO-1, which has not been reported before.

### AUY922 inhibits the NF-kB, NFAT and AP-1 signal transduction pathways, and this phenotype is recapitulated by targeting TAK1

**Fig 4:**
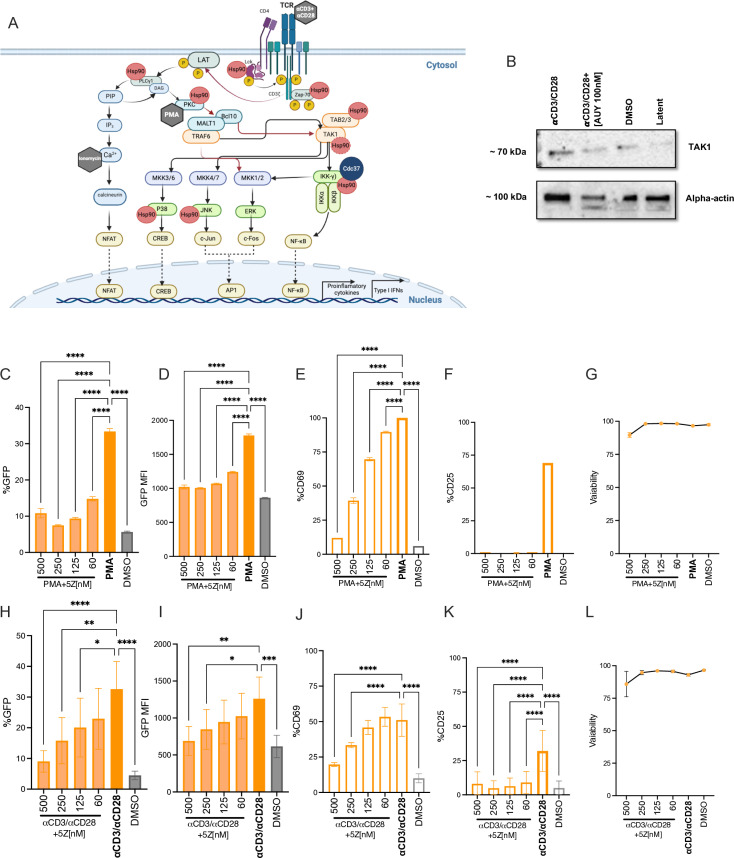
The TAK1 inhibitor 5Z mimics the effects of AUY922. (A) Schematic description of the TCR signal transduction pathways and the factors that are targeted by the LRAs (grey hexagon) or that are known Hsp90 client proteins (red circles). (B) Western blot to detect total TAK1 in latently infected Jurkat cells. Each lane was loaded with an equal number (1x10^6^) of lysed cells. (C-F) Latently infected Jurkat cells were stimulated with PMA [0.9ng/ml] for 24 hours in the presence of the indicated concentrations of TAK1 inhibitor 5Z and analysed by flow cytometry. (C) Bar graphs show the average percentage ± SD of GFP+ cells, (D) average GFP MFI ± SD, (E) average percentage ± SD of CD69+ cells, (F) average percentage ± SD of CD25+ cells and (G) cell viability. (H-K) Latently infected Jurkat cells were stimulated with anti-CD3/CD28 Abs [1μg/ml/2μg/ml] in the presence of the indicated concentrations of 5Z for 24 hours and analysed by flow cytometry. (H) Bar graphs showing average percentage ± SD of GFP+ cells, (I) average GFP MFI ± SD, (J) average percentage ± SD of CD69+ cells, (K) average percentage ± SD of CD25+ cells and (L) cell viability. N = 6. Statistical significance was calculated using one-way ANOVA with Dunnett’s correction. *=p≤0.05; **=p≤0.01; ***=p≤0.001; ****=p<0.0001. Schematic in (A) was created with BioRender.com.

The broad activity of AUY922 supported the notion that Hsp90 regulates multiple signalling cascades important for HIV-1 reactivation. Stimulation of CD4+ T cells by anti-CD3/CD28 antibodies induces the NF-kB, NFAT and the AP-1 (c-Jun and c-Fos) signal transduction pathways. The TCR activates PLCγ1 (an Hsp90 client protein) and generates DAG and IP₃ through the hydrolysis of PIP(4,5)P₂. DAG activates protein kinase C (PKC), also activated by PMA, triggering a signalling cascade that eventually activates NF-kB and AP-1, while IP₃ increases intracellular Ca²⁺ levels, which activate calcineurin. Calcineurin dephosphorylates NFAT, enabling its nuclear translocation [59,60] (Fig 4A). NF-kB, AP-1 and NFAT are key regulators of HIV-1 gene expression [19]. The NF-kB and AP-1 signalling pathways share a key upstream regulator called transforming growth factor-β–activated kinase 1 (TAK1) [59,61–66] (Fig 4A). TAK1 is a Hsp90 client protein [67,68] and has also been reported to bind to the accessory HIV1 protein Vpr, which appears to enhance TAK1 activity [69].

We therefore asked if TAK1 contributes to HIV-1 reactivation through Hsp90. When Hsp90 is inhibited, there is a loss of total TAK1, which is degraded [67,68]. Hence, we have examined total TAK1 by Western blot in our latent Jurkat cells before and after stimulation with anti-CD3/CD28 Abs, with or without AUY922. Our results showed that TCR stimulation increases total TAK1, which was reduced in the presence of AUY922, in agreement with the earlier reports (Fig 4B) [67,68]. Next, we stimulated the latent Jurkat cells with a fixed concentration of PMA for 24 hours in the presence of 5*Z*-7-Oxozeaenol (5Z), a selective inhibitor that blocks the ATPase activity of TAK1 [70]. Cells were analysed by flow cytometry to measure the percentage of GFP+ cells, GFP MFI, CD69, CD25 and cell viability. Treatment with PMA triggered viral reactivation, which was repressed by 5Z in a dose dependent way, both in terms of percentage of GFP+ cells and GFP MFI (Fig 4C and 4D). Surface expression of activation markers CD69 and CD25 was also significantly inhibited by 5Z (Fig 4E and 4F) in the absence of detectable cell toxicity (Fig 4G). Similar results were obtained following TCR stimulation of the latently infected cells (Fig 4H–4L). Hence 5Z appeared to mimic the effect of AUY922 on HIV-1 reactivation.

**Fig 5:**
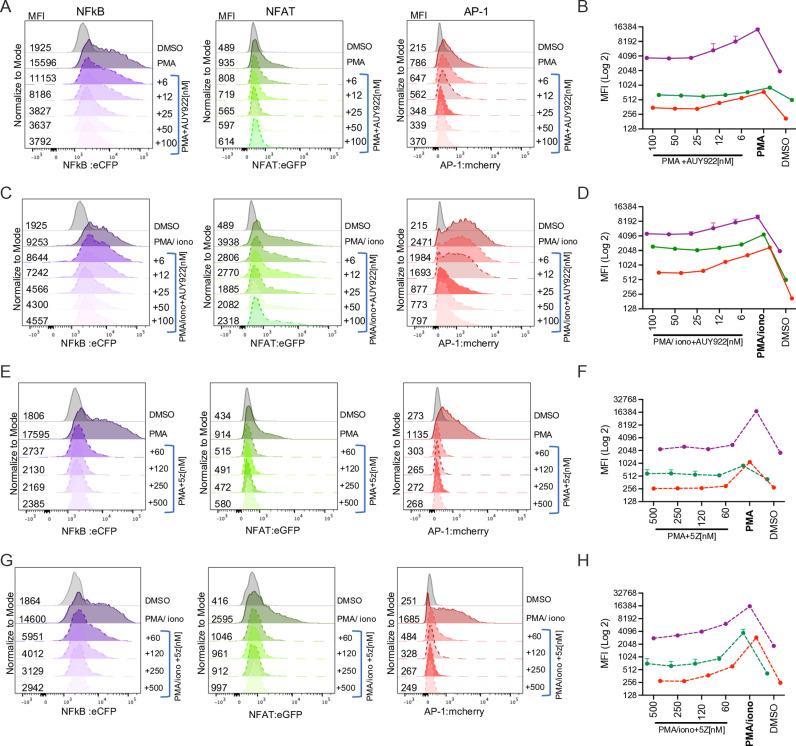
AUY922 and 5Z suppress NF-kB, AP-1 and NFAT. (A) TPR cells were stimulated with PMA [125 ng/ml] in the presence of the indicated concentrations of AUY922. MFI was measured by flow cytometry to detect activation of NF-kB (left panel), NFAT (middle panel) and AP-1 (right panel). (B) Graph showing average MFI values ± SD (on a Log2 scale) for each factor, (n= 6). (C) TPR cells were stimulated with PMA [125 ng/ml] + ionomycin [4 μg/ml] in the presence of the indicated concentrations of AUY922 and analysed by flow cytometry as in (A). (D) Graph showing average MFI values ± SD (on a Log2 scale) for each factor. (E) TPR cells were stimulated with PMA [125 ng/ml] in the presence of the indicated concentrations of 5Z and analysed by flow cytometry as in (A). (F) Graph showing average MFI values ± SD (on a Log2 scale) for each factor. (G) TPR cells were stimulated with PMA [125 ng/ml] + ionomycin [4 μg/ml] in the presence of the indicated concentrations of 5Z and analysed by flow cytometry as in (A). (H) Graph showing average MFI values ± SD, n = 6 (on a Log2 scale) for each factor.

We sought to test the effect of AUY922 and 5Z on the individual transcription factors NF-kB, AP-1 and NFAT. To this end, we took advantage of the triple parameter reporter (TPR) indicator cells [71], a Jurkat cell line in which the response elements for NF-κB, NFAT and AP-1 drive the expression of the fluorescent proteins eCFP, eGFP and mCherry, respectively, to simultaneously detect by flow cytometry the activation of these transcription factors after stimulation [71]. TPR cells were stimulated as before with a fixed concentration of PMA in the presence of AUY922 and analysed by flow cytometry after 24 hours. As expected, PMA triggered robust activation of NF-kB and AP-1, and a more modest activation of NFAT, and AUY922 reduced this effect in a dose dependent way (Fig 5A and 5B). Stimulation with ionomycin alone did not trigger activation of the transcription factors (S5A and S5B Fig) but the combination of PMA + ionomycin enhanced the activation of NFAT and AP-1 above PMA alone, although NF-kB remained unaffected and AUY922 counteracted this effect in a dose-dependent way (Fig 5C and 5D). These results confirmed that Hsp90 is critical for the activation of several key transcription factors involved in HIV-1 latency. Of note, the inhibitory effect of AUY922 appeared to be weaker for NFAT relative to NF-kB and AP-1 and to reach a plateau between 25 and 50 nM, suggesting that activation of the NF-kB, AP-1 and NFAT signal transduction pathways does not rely exclusively on Hsp90, which may explain the lack of cell toxicity at the tested concentrations. Our TPR cells lacked the endogenous TCR (α-chain), hence, to test the effect of TCR stimulation, we employed a TPR derivative in which the α-chain was lentivirally-transduced. In these cells, stimulation with anti-CD3/CD28 antibodies triggered activation of NF-kB and AP-1 and this effect was reduced by AUY922, whereas the drug did not abrogate the induction of NFAT (S6A and S6B Fig).

We repeated these experiments in the presence of the TAK1 inhibitor 5Z, and the results showed a dose-dependent repression of NF-kB and AP-1 following stimulation with PMA (Fig 5E and 5F). NFAT was less sensitive to 5Z (Fig 5F), which mimicked the observations with AUY922 (Fig 5B). Stimulation of the cells with PMA + ionomycin enhanced NFAT activity well above PMA alone, and a significant proportion of the signal was reduced by 5Z in a dose-dependent way (Fig 5G and 5H). 5Z also reduced the activation of NF-kB, AP-1 and NFAT triggered by TCR stimulation (S6C and S6D Fig). The mechanism by which 5Z reduces NFAT activation in the TPR cells remains unclear, as TAK1 is not directly linked to the NFAT signalling pathway. However, there is evidence for a crosstalk between TAK1 and the calcineurin-NFAT pathway through its interaction with RCAN1 (Regulator of Calcineurin 1), a key modulator of this pathway [72]. Taken together, these results indicate that pharmacological inhibition of Hsp90 and TAK1 have a similar effect on HIV-1 reactivation, supporting the idea that TAK1 is a Hsp90 chaperone that may contribute to HIV-1 reactivation. However, we cannot exclude that 5Z mimicked AUY922 by a different mechanism.

### Short-term inhibition of Hsp90 does not affect the phenotype of stimulated primary CD4+ T cells

For clinical applications, agents that suppress HIV-1 reactivation should minimally affect the phenotype and function of the host CD4+ T cells. We therefore investigated if inhibiting Hsp90 changed the phenotype and differentiation of primary CD4+ T cells after stimulation. To this end, CD4+ T cells were isolated by magnetic sorting from peripheral blood mononuclear cells (PBMCs) of healthy volunteers and incubated for 72 hours with IL-2 only (No stim), or anti-CD3/CD28 antibodies plus IL-2 to induce TCR stimulation, or anti-CD3/CD28 antibodies plus IL-2 and 25 nM AUY922, which was added 48 hours after stimulation. Cells were then analysed by spectral flow cytometry using a 18-colour antibody panel (S3 Table) [73–79] designed to detect four main CD4+ T cell subsets: naïve (CD3+CD4+, CD45RA+, CCR7+), T central memory (Tcm) (CD3+CD4+, CD45RA-, CCR7+, CD45RO+), T effector memory (Tem) (CD3+CD4+, CD45RA-, CCR7-, CD45RO+), T effector (CD3+CD4+, CD45RA+, CCR7-), and subsets Th17 (CD194+, CD196+), Th1 (CD194- and CD183+), and Th2 (CD194+, CD196-). The activation state of the cells was examined using activation markers CD25, CD69, HLA-DR and CD38 and inhibitory markers PD-1, TIGIT, and Tim-3 (S3 Table) [73,80–83]. To precisely gate the correct population, Fluorescence Minus One (FMO) gating was performed for each of the antibodies on stimulated cells with no AUY922 (S7A Fig). The gating strategy is described in (S7B Fig). The parameters set for this FMO staining were subsequently used to gate the samples.

**Fig 6:**
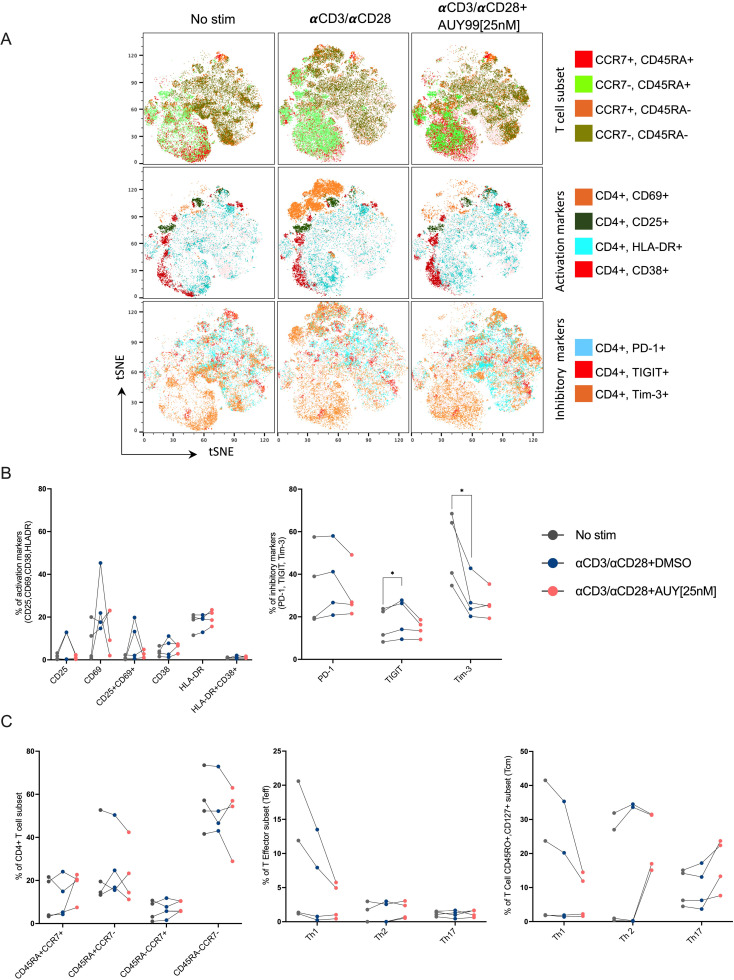
The effect of AUY922 treatment on different CD4+ T cell populations. Primary CD4+ T cells were treated with IL-2 only (No stim) or with anti-CD3/CD28 Abs and IL-2 for 72 hours, and AUY922 [25 nM] or DMSO was added at 48 hours post-stimulation. Cells were analysed by spectral flow cytometry using a panel of 18 antibodies 24 hours after the addition of AUY922. (A) Representative tSNE plot for one donor is shown. (B-C) Results from four donors are shown. Cell subsets were identified according to the combination of surface markers shown in S4 Table and the gating strategy shown in S7 Fig. Statistical significance was calculated using two-tailed paired t-Test: *=p≤0.05; **=p≤0.01; ***=p≤0.001; ****=p<0.0001.

The flow cytometry high dimensional data were analysed by automated t-distributed stochastic neighbour embedding (tSNE) [84]. The tSNE plots showed no appreciable changes in the main cell populations, including samples treated with the Hsp90 inhibitor (Figs 6A and S8). Notably, we detected a population of CD45RA+ CCR7+ “naïve” cells despite TCR stimulation, which indicates that treatment with anti-CD3/CD28 antibodies did not uniformly activate all the cells. Expression of the activation markers CD69 and CD25 was higher in stimulated cells and AUY922 reduced CD69 levels in 2 out of 4 donors. No changes were observed for the late stimulation markers HLA-DR and CD38 but treatment with anti-CD3/CD28 Abs upregulated TIGIT and downregulated Tim-3, which then remained unaffected by AUY922 (Figs 6A, 6B and S8). We then quantified the flow cytometry results and performed statistical analysis, which confirmed that AUY922 treatment did not significantly change the activation state of the CD4+ T cells or the subtypes frequency, although we noted a trend for fewer CD25+ cells (Fig 6B).

### Inhibition of Hsp90 suppresses HIV-1 reactivation without affecting the differentiation phenotype of primary CD4+ T cells

**Fig 7:**
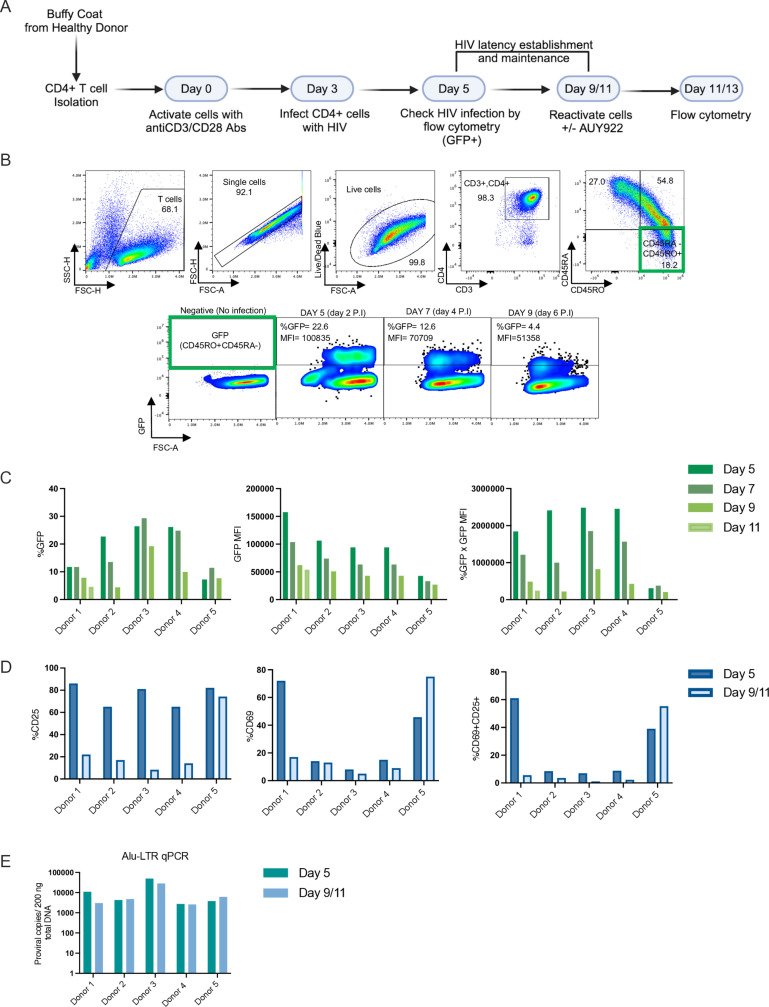
Generation of the ex-vivo latency infected primary CD4+ T cells. (A) Schematic depiction of the experimental set-up to generate latently infected cells ex-vivo. Cells were infected at day 3 post-stimulation with a VSV-G pseudotyped, single cycle HIV-1 reporter virus (pNL4-3-Δ6-drEGFP). (B) Representative flow cytometry plots showing the gating strategy used to detect the GFP+ cells in the CD45RA- CD45RO+ memory population. Cells were analysed by flow cytometry for GFP expression at regular intervals. (C) Bar graphs showing the percentage of GFP+ cells (left panel), the GFP MFI (middle panel), and combined % GFP x MFI (right panel) measured for each donor on the indicated days. (D) Bar graphs showing the percentage of CD25+ cells (left panel), CD69+ cells (middle panel), and double CD25+CD69+ cells (right panel) for each donor measured by flow cytometry on the indicated days. (E) DNA was extracted from the CD4+ T cells on the indicated days and used to quantify integrated proviral DNA copies by Alu-LTR qPCR. Schematic in (A) was created with BioRender.com.

These results indicated that inhibition of Hsp90 by AUY922 at 25nM did not appreciably alter the overall phenotype of uninfected and stimulated CD4+ T cells (Fig 6). Next, we examined the selectivity of AUY922 in a primary model of HIV-1 latency. To this end, CD4+ T cells were isolated from PBMCs of 5 healthy donors, which were stimulated in vitro by treatment with anti-CD3/CD28 antibodies in the presence of IL-2 for 3 days, after which cells were infected with the single cycle NL4.3Δ6-drGFP virus. Two days later, an aliquot of the cells was analysed by flow cytometry to measure the percentage of GFP+ cells and GFP MFI. Infected cells were maintained in culture with IL-2 for up to 11 days and regularly monitored by flow cytometry to assess the establishment of latency (Fig 7A and 7B). HIV-1 latency manifests as reduction of viral gene expression, best captured in our model by measuring GFP MFI, and also as a complete loss of viral gene expression in individual cells, best captured by measuring the percentage of GFP+ cells. We therefore used both measurements and a combination thereof to assess latency and reactivation in the primary model. In the latency phase, there was a progressive loss of viral gene expression, which was more marked when measured as GFP MFI than percentage of GFP+ cells (Fig 7C). Surface expression of CD69+ and CD25+ was also measured to monitor the T cell activation, which showed that infected cells returned to a more resting state over time, except for donor 5 (Fig 7D). In parallel, we measured proviral DNA copy number by Alu-LTR qPCR [85] at day 5 and between day 9 and day 11 and found little or no loss of proviral DNA, confirming that genuine latency was established in the primary CD4+ T cell model (Fig 7E).

**Fig 8:**
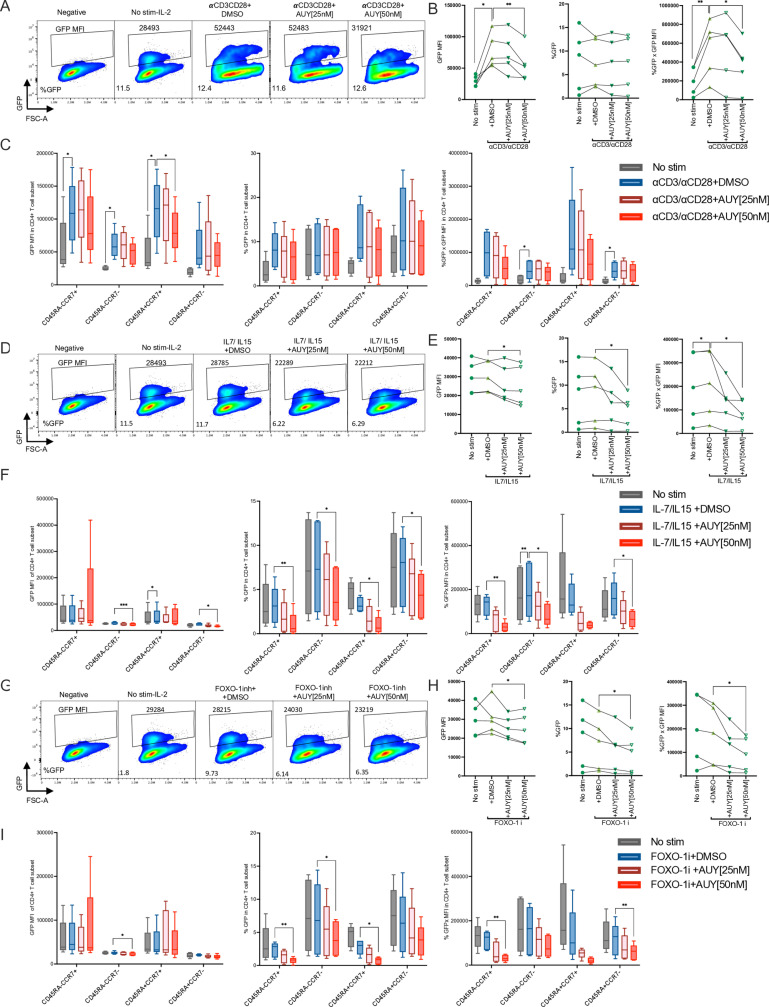
Effect of AUY922 on HIV-1 reactivation induced by different stimuli in primary CD4+ T cell subsets. Latently infected primary CD4+ T cells were generated ex vivo, as described in Fig 7A. Cells were re-stimulated on day 9 or 11 with the indicated LRA with or without AUY922 [25 nM or 50 nM] or DMSO. Cells were analysed by spectral flow cytometry using the same antibody panel used in Fig 6, in addition to GFP. (A) Representative flow plots showing the GFP MFI before (No stim-IL-2) and after re-stimulation with anti-CD3/CD28 antibodies with or without AUY922 [25 or 50 nM]. (B) Graphs showing the results for 5 donors: GFP MFI (left panel), percentage of GFP+ cells (middle panel), and combined % GFP x MFI in the CD45RA- CD45RO+ population. (C) Results from five donors are shown for the GFP MFI (right panel), percentage of GFP+ cells (middle panel) and combined % GFP x MFI (right panel) in each CD4+ T cell subset. Bar plots show 1st quartile, 3rd quartile and median for five donors. (D) Representative flow cytometry plots showing the GFP MFI before (No stim-IL-2) and after re-stimulation with IL-7 + IL-15 [20 ng/mL each] with or without AUY922. (E) Graphs showing the results for 5 donors: GFP MFI (left panel), percentage of GFP+ cells (middle panel), and combined % GFP x MFI in the CD45RA-CD45RO+ population. (F) Same as described in (C). (G) Representative flow plots showing the GFP MFI before (No stim-IL-2) and after re-stimulation with the FOXO-1 inh [200 nM] with or without AUY922. (H) Graphs showing the results for 5 donors: GFP MFI (left panel), percentage of GFP+ cells (middle panel), and combined % GFP x MFI in the CD45R-CD45RO+ population. (I) Same as described in (C). Statistical significance was calculated using a two-tailed paired t-Test comparing anti-CD3CD28 versus no stim-IL-2, and anti-CD3CD28 versus 50 nM AUY922, *=p≤0.05; **=p≤0.01; ***=p≤0.001; ****=p<0.0001.

To induce viral reactivation, cells were exposed to anti-CD3/CD28 antibodies, IL-7/IL-15 or the FOXO-1 inhibitor in the presence of absence of AUY922 (25 or 50nM) or DMSO for 48 hours. Treated cells were analysed by spectral flow cytometry as described in S7 Fig to measure the percentage of GFP+ cells and its MFI both in the general population and in each CD4+ T cell sub-type. In CD45RA- CD45RO+ memory cells, anti-CD3/CD28 antibodies triggered significant reactivation, as measured by the GFP MFI, which was inhibited by AUY922 in a dose-dependent way (Fig 8A and 8B). TCR stimulation did not appear to appreciably increase the percentage of GFP+ cells, nonetheless when both the GFP MFI and the percentage of GFP+ cells were combined, TCR stimulation was found to be effective, and this effect was partially abrogated by AUY922 at 50nM (Fig 8B). We then assessed HIV-1 reactivation and the effect of AUY922 in the other CD4+ T cell subsets. Within specific CD4+ T cell populations, CD45RA+ CCR7+ “naïve” cells showed the greatest susceptibility to AUY922, followed by CD45RA- CCR7+ central memory cells [86] (Figs 8C and S9A and S9B), although the latter trend did not reach statistical significance.

Cells treated with IL-7 and IL-15 did not show appreciable reactivation above IL-2 only (No stim.), however inhibition of Hsp90 significantly reduced viral gene expression below the baseline (unstimulated cells) in CD45RA- CD45RO+ memory cells and this effect was significant for GFP MFI, percentage of GFP+ cells and the combination of GFP MFI and % GFP+ cells (Fig 8D and 8E). Within specific CD4+ T cell subsets, the greatest susceptibility to AUY922 was detected in CD45RA+ CCR7+ cells, followed by CD45RA- CCR7+ cells, and CD45RA- CCR7- effector memory cells (Figs 8F and S9C). Treatment with the FOXO-1 inhibitor failed to induce viral reactivation but inhibition of Hsp90 significantly reduced viral reactivation below the baseline of unstimulated CD45RA- CD45RO+ cells (Fig 8G and 8H). In the FOXO-1 inhibitor-treated cells, we detected the strongest response to AUY922 in the CD45RA- CCR7+ and CD45RA+ CCR7- populations, followed by the CD45RA+ CCR7+ population (Figs 8I and S9D).

It is not clear why IL-7 + IL-15 and the FOXO-1 inhibitor did not trigger appreciable viral reactivation however we note that the conditions of the experiments on Jurkat cells and on primary CD4+ T cells were different, mainly because the primary cells had been pre-stimulated with anti-CD3/CD28 antibodies to make them permissive to HIV-1 infection 10 days before re-stimulation, which might have affected their response to the LRAs themselves. The presence of IL-2 at all stages was essential for cell survival in our experimental conditions and so we were unable to control for its effects. However, we found that treatment with AUY922 did not reduce baseline HIV-1 gene expression in the presence of IL-2 alone (S10 Fig). Thus, background activity of IL-2 might have partially masked the reactivating effect of IL-7/IL-15 and the FOXO-1 inhibitor, but the effect of AUY922 appeared to depend on the addition of these LRAs. Furthermore, the threshold for the response to LRAs might be different between Jurkat and primary cells. Nonetheless, the results confirmed that targeting Hsp90 reduced TCR-induced reactivation and inhibited baseline viral gene expression in latently infected cells even in the presence of IL-7 and IL-15 or the FOXO-1 inhibitor.

**Fig 9:**
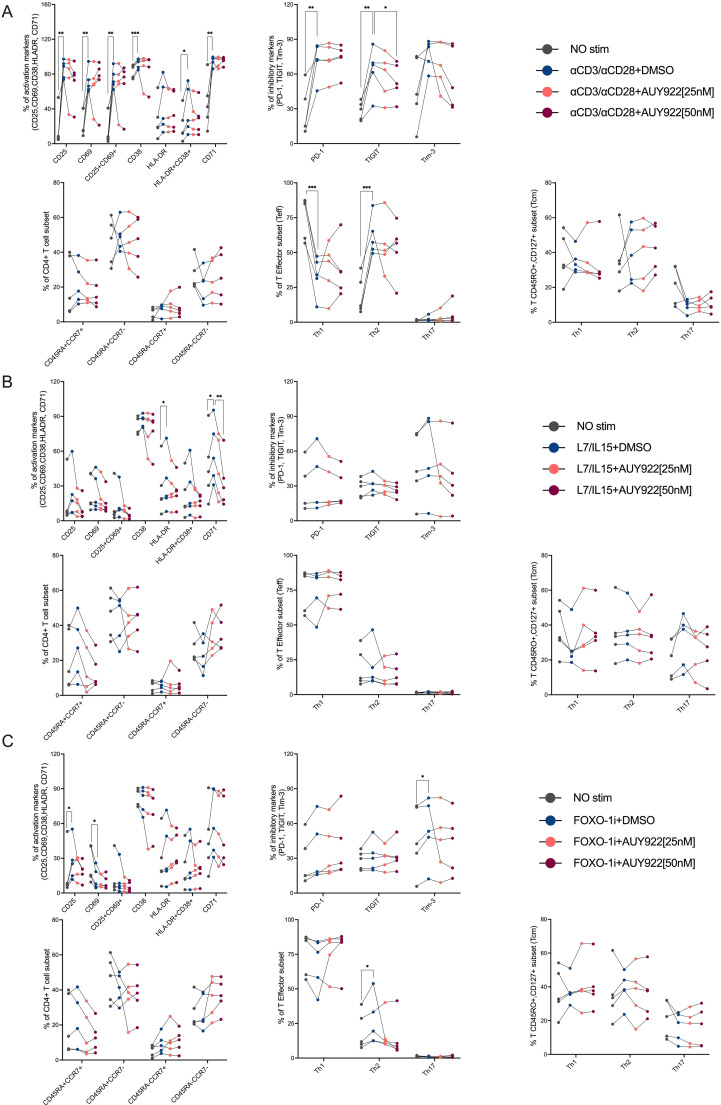
AUY922 does not significantly change the CD4+ T cell phenotype or activation state. Latently infected primary CD4+ T cells were re-stimulated on day 9 or 11 with the indicated LRA with or without AUY922 [25 nM or 50 nM] or DMSO. Cells were analysed by spectral flow cytometry using the same antibody panel used in Fig 6 with the addition of CD71. (A) panels show, in this order from left to right, top to bottom: T cell activation markers, inhibitory markers, CD4 T cell subsets, effector subsets and memory subsets after re-stimulation with anti-CD3 [1 µg/ml] and anti-CD28 [2 µg/ml] antibodies plus AUY922 [25 or 50 nM] or DMSO. (B) Same measurements for cells re-stimulated with IL-7/IL-15 [20ng/mL]. (C) Same measurements for cells restimulated with FOXO-1 inh. [200nM]. Results were obtained from five different donors. Statistical significance was calculated using a two-tailed paired t-Test comparing, within each cell sub-type, anti-CD3CD28 versus no stim (IL-2), anti-CD3CD28 versus 50 nM AUY922, *=p≤0.05; **=p≤0.01; ***=p≤0.001; ****=p<0.0001.

We also examined if inhibition of Hsp90 at levels sufficient to reduce viral gene expression affected the activation phenotype of CD4+ T cells. In cells stimulated twice by anti-CD3/CD28 antibodies, we found a significant upregulation of activation markers CD69, CD25, CD38 and CD71 (the transferrin receptor) [87] with simultaneous upregulation of inhibitory markers PD-1, TIGIT and Tim-3 (the latter showed a trend but did not reach statistical significance), which suggested a degree of exhaustion after two rounds of stimulation in a relatively short time interval [81] (Fig 9A). Treatment with AUY922 did not appreciably affect expression of these markers, except for a reduction in TIGIT surface expression (Fig 9A). We also found a significant reduction in the proportion of markers of Th1 cells against a significant increase in the proportion of markers of Th2 cells but treatment with AUY922 did not change this (Fig 9A). Treatment with IL-7/IL-15 significantly upregulated HLA-DR and CD71, confirming activity of the cytokines, and AUY922 significantly reduced expression of CD71 (Fig 9B). The FOXO-1 inhibitor significantly upregulated CD25, downregulated CD69 and showed a trend for CD71 upregulation (Fig 9C). These results indicated that IL-7/IL-15 had biological activity on the CD4+ T cells, even if they did not appear to stimulate HIV-1 reactivation above IL-2 alone. Therefore, in our experimental conditions, inhibition of Hsp90 did reduce HIV-1 gene expression without any apparent significant effect on the differentiation and phenotypic markers of CD4+ T cells.

Lastly, we investigated if treatment with AUY922 affected cytokines production from activated CD4+ T cells. To this end, we collected the supernatant from the cell cultures of the same 5 donors described in Fig 8B. Supernatants were collected 48h post-restimulation with anti-CD3/CD28 Abs to measure the concentration of 5 cytokines that define specific subsets of effector CD4+ T cells: IFNγand TNF-α (Th1), IL-4 and IL-10 (Th2) and IL-17A (Th17) [88]. TCR stimulation significantly increased production of all tested cytokines, however AUY922 significantly but modestly inhibited only IL-4 and IL-10 production (S11 Fig). There was a downward trend for the other cytokines in the AUY922-treated samples compared to untreated, but it did not reach statistical significance. These results confirmed that inhibition of Hsp90 did not broadly perturb CD4+ T cell function, although it might modestly reduce production of Th2 and Th1 cytokines.

## Discussion

HIV-1 latency is regulated at multiple levels, which include the epigenetic features of chromatin at the proviral integration site, the availability of specific host cell factors that activate viral transcription, the post-translational regulation of such host factors and the intactness of the provirus itself [18,19]. The mechanisms that influence HIV-1 latency, or its reactivation, are intimately linked to the activation and differentiation of the infected CD4+ T cells [19]. Therefore, strategies to either enhance HIV-1 reactivation from latency or induce a deeper state of latency need to address two problems: the multi-factorial nature of latency and simultaneously interfering as little as possible with the function and differentiation of the infected cells.

Here, we have confirmed that inhibiting Hsp90 antagonises HIV-1 reactivation triggered by LRAs such as TCR, PMA, PHA and TNF-α[13,89] and showed, for the first time to our knowledge, that Hsp90 is also required for HIV-1 reactivation induced by TLR7 and TLR8 stimulation and FOXO-1 inhibition. No significant loss of cell viability was detected in our experimental conditions and similar results were obtained using AUY922 or 17-AAG, two structurally different Hsp90 inhibitors that bind to the same ATPase pocket in the N-terminal region of the chaperone, supporting the specificity of the effect. AUY922 was chosen for our experiments because it is a well-characterized drug that has been used in phase II and III clinical trials [90].

The repressive effect of AUY922 was broad and extended to many pathways that are physiologically involved in HIV-1 reactivation [91,92]. This can be explained by the numerous client proteins that depend on Hsp90 for correct folding and assembly, which are also key components of the signalling pathways critical for virus reactivation [93]. We and others have previously shown that Hsp90 is part of the IKK complex and promotes its activity, and that it stabilizes P-TEFb [13,30]. Here, we have extended these observations and shown that Hsp90 is critical for activation of the NF-kB, and AP-1 signal transduction pathways and to a lesser extent the NFAT pathway, supporting the notion that the chaperone controls multiple key events in viral reactivation. Notably, we found that AUY922 reduced total TAK1 in stimulated cells and that the TAK1 antagonist 5Z phenocopied the effect of AUY922 in latently infected cells, suggesting that at least part of AUY922 anti-reactivation effect may be mediated by TAK1. However, we cannot exclude that 5Z exerted its effects by inhibiting additional unknown targets.

Hence short-term inhibition of Hsp90 appears to address, albeit not fully, the first problem related to the multifactorial nature of HIV-1 latency, and in this respect Hsp90 may be considered a master regulator. But does inhibition of Hsp90 addresses the second problem related to the effect of latency potentiating agents on the physiology of the infected CD4+ T cells?

To answer this, we have initially examined the effect of AUY922 on the phenotypic profile of uninfected CD4+ T cells. A well-established method to study the profile of T cells and their differentiation is multiparameter flow cytometry, which measures the combinatorial expression of chemokine receptors and other differentiation markers on the cell surface [73]. Surface expression of these markers is tightly regulated at the transcriptional and post-transcriptional levels and reflects the overall physiological state of the cell [94,95]. We applied a carefully selected panel of 18 markers that, when suitably combined, define several subsets of CD4+ T cells, including naïve, effector, central memory, effector memory and subtypes Th1, Th2 and Th17. We have also employed surface markers of activation, whose expression changed depending on the applied LRA in agreement with its known physiological effect. The results of these experiments revealed no significant change in any of the cell phenotypes, suggesting that the degree of Hsp90 inhibition applied to inhibit HIV-1 reactivation was well tolerated.

Next, we assessed the impact of AUY922 on infected CD4+ T cells. We generated ex-vivo primary latently infected cells, which required a first round of T-cell stimulation to make cells permissive to HIV-1 infection, passaging the cells to allow them to return to a more quiescent state and establish viral latency, and a second round of stimulation to trigger viral reactivation in the presence of AUY922. We were able to establish viral latency in this model, although there was donor-to-donor variability in the magnitude of reactivation. AUY922 antagonized viral reactivation and reduced the level of viral gene expression as measured by the GFP MFI, although it did not seem to fully block it, which would have resulted in a lower percentage of GFP+ cells. This differed from Jurkat cells, in which AUY922 reduced both GFP MFI and percentage of GFP+ cells, suggesting greater potency of the drug. This discrepancy between primary CD4+ T cells and Jurkat cells may be due to the well-known heightened sensitivity of cancer cells to Hsp90 inhibitors [96,97]. The heightened sensitivity of cancer cells to AUY922 and other Hsp90 inhibitors has been linked to the greater dependency of cancer cells on the chaperone due to higher protein synthesis and metabolism, and possibly to a conformation of Hsp90 that seems better targeted by drugs in cancer cells, but this latter possibility is controversial [96].

The combination of IL-7 and IL-15 did not reactivate HIV-1 in our primary model or in Jurkat cells. IL-7 renders resting CD4+ T cells more permissive to HIV-1 infection [98] through the Jak/STAT5 pathway [99] and by partially activating the cells [100]. IL-7 has also been reported to activate latent HIV-1 ex-vivo in human CD4+ T cells from humanized mice [101], and in cells from patients at the same concentrations we tested, however this effect was reported to be variable from donor to donor, proviral-strain specific and was seen after several days of exposure to the cytokine [41,102]. Furthermore, significant IL-7 induced reactivation is not universally observed [103,104]. We detected upregulation of HLA-DR, as previously described [102], and CD71 upon treatment with IL-7/IL15, but we did not detect viral reactivation. We surmise that the difference with some earlier studies may be due to the shorter duration of treatment in our experiments relative to other studies [41,102]. It is also possible that IL-2, by partly stimulating HIV-1 gene expression, masked the activating effect of IL-7/IL-15. Notably, AUY922 suppressed viral gene expression below the baseline even in the presence of IL-7/IL-15, confirming its activity on the HIV-1 promoter [13].

Treatment of the cells with the FOXO-1 inhibitor upregulated surface expression of CD71, in agreement with previous reports [52] but induced weak reactivation in two out of five donors. This result contrasted with the reproducible reactivation that we and others found in Jurkat cells [51,52] and we speculate that this difference in the response is due to the short time of exposure to the FOXO-1 inhibitor. Regrettably, our primary latency model did not allow to extend the duration of the experiment beyond 12–13 days because of a notable drop in cell viability after that time point. Nonetheless, even in the presence of the FOXO-1 inhibitor, AUY922 reduced viral reactivation below the baseline of untreated cells.

Different cell subsets showed different responses to the LRAs and AUY922. Overall, the strongest viral reactivation after TCR stimulation and the greatest susceptibility to AUY922 was detected in CD45RA+ CCR7+ cells, which is largely made of naïve cells but can also contain a small proportion of T memory stem cells (Tscm) [105] followed by CD45RA- CCR7+ central memory cells, and CD45RA- CCR7- effector memory cells, which agrees with a previous study [47]. Certain CD4+ T cell subsets have been shown to be more susceptible to specific LRAs but the reasons for this behaviour are not clear [106–109]. Our results extend these observations to include CD4+ T cell subtype-specific responses to Hsp90 inhibition and it will be interesting to investigate the mechanistic reason for this phenotype and how different LRAs and Hsp90 intersect in the different CD4+ T cell subtypes.

Notably, treatment with AUY922 did not significantly change the proportion of each CD4+ T cell subtype in the population and had a modest effect on Th2 and Th1 cytokine production, which demonstrated that inhibiting Hsp90 does reduce HIV-1 reactivation without dramatically altering CD4+ T cell differentiation or activation state, at least in the experimental conditions tested. The weak inhibition of Th1 cytokine production may be advantageous in PLWH, who often have heightened general inflammation. We note that TCR stimulation enhanced surface expression of activation and exhaustion markers and reduced the relative proportion of the markers for Th1 cells, indicating that our readout was able to detect phenotypic changes in the samples. Considering that Hsp90 participates in a wide variety of cellular pathways, these results may seem surprising. Selectivity is usually a function of the drug concentration and time of exposure, which in our case was limited to 48 hours. It is possible that a longer incubation time in the presence of the drug may affect the phenotype of CD4+ T cells, and it should be possible to evaluate this aspect in patients who are being treated with AUY922 or other Hsp90 inhibitors in clinical trials [110].

Hsp90 inhibitors are being evaluated in clinical trials for the treatment of both solid and haematological malignancies, at concentrations that are significantly higher than those that are sufficient to repress HIV-1 gene expression [90]. Although so far Hsp90 inhibitors have not shown good efficacy above baseline for the treatment of solid cancers [110,111], their pharmacological and toxicity profiles are well-known. It would therefore be conceivable to assess Hsp90 inhibitors in the context of analytical treatment interruption (ATI) studies to test if virological rebound is delayed with the view of using the inhibitors in conjunction with other eradication strategies, especially if the long-lasting repression of virological rebound reported in humanized mice are reproduced in patients [28]. Of note, 17-AAG was recently shown to significantly reduce HIV-1 reactivation in latently infected patients’ cells [112]. An interesting question to address in future studies will be whether inhibition of Hsp90 synergises with the tat inhibitor dCA [24,25] or with AhR agonists [21] to stably repress HIV-1 reactivation.

Lastly, Hsp90 is required for the replication of many RNA and DNA viruses [113], including early gene expression of human cytomegalovirus (HCMV) [114] and it could therefore be a broad antiviral target for the treatment of co-infections, often seen in PLWH [115].

Our study has some limitations. The Jurkat and primary cell models of latency may not fully recapitulate the complexity of the in vivo latent reservoir and the viral construct used does not express accessory proteins Vpr and Vpu, which may have a role in latency and viral gene expression [116,117]. Although we observed clear trends in the primary cell model, not every result reached statistical significance due to donor-to-donor variability in the degree of viral reactivation and this issue will need to be carefully considered in future pre-clinical studies.

## Materials and methods

### Ethics statement

Blood samples were obtained from healthy volunteers after written informed consent, or from the National Health Service Blood and Transplant (NHS-BT) according to the approved protocol of the University College London Research Ethics Committee reference REC 3138/001 and NHS-BT reference R140.

### Chemical compounds

NVP-AUY922 was purchased from LKT Laboratories; 17-allylamino-17-demethoxygeldanamycin (17-AAG or tanespimycin) from Merck Life Science UK Ltd. Phorbol 12-myristate 13-acetate (PMA), 5*Z*-7-Oxozeaenol (5Z) were obtained from Cayman Chemical; TNF-α was purchased from Life Technologies Limited. Imiquimod (TLR7 agonist) was purchased from Alfa Aesar, CL075 (TLR8 agonist) from Sigma-Aldrich (MREK). FOXO-1 inh (AS1842856) was purchased from MedChemExpress LLC, Phytohemagglutinin (PHA) and Ionomycin were obtained from Thermo Fisher Scientific. Compounds were dissolved in DMSO to obtain 1000x stock solutions (v/v) and stored in aliquots at −20 °C in the dark. Purified anti-human CD3 antibody (OKT) and anti-human CD28 antibody (CD28.2) were purchased from Biolegend; Recombinant human IL-7, rhIL-2, IL-6 and IL-15 were obtained from Peprotech. The concentrations used are shown in S2 Table for each compound.

### Cells and tissue culture

The Jurkat cell line E6-1 was obtained from the American Type Culture Collection (ATCC) and cultured in RPMI media (Gibco) supplemented with 10% fetal bovine serum, 100 U/mL penicillin/streptomycin, and maintained at 37°C in a 5% CO₂ incubator. 293T cells were obtained from the ATCC and maintained in DMEM media (Gibco) supplemented with 10% fetal bovine serum, 100 U/mL penicillin/streptomycin. Peripheral blood mononuclear cells (PBMCs) were isolated by Ficoll-Paque PLUS density gradient centrifugation. Human CD4+ T lymphocytes were isolated from PBMCs using the MojoSort Human CD4+ T Cell Isolation Kit (BioLegend, 480010) following the manufacturer’s instructions. Isolated CD4+ T cells were cultured in X-VIVO 15 Serum-free Hematopoietic Cell Medium (Lonza), supplemented with 5% fetal bovine serum (FBS; Labtech) and 100 U/mL penicillin/streptomycin. Triple parameter reporter (TPR) Jurkat-derived cells were kindly provided by Prof. Peter Steinberger (Medical University of Vienna) and cultured in RPMI as above.

### Virus production

The single-cycle HIV-1 vector (Δenv) was produced by transfection of 293T cells using FuGENE Transfection Reagent as previously described [22,45]. The transfection mixture included the following plasmids: pNL4–3-Δ6-drEGFP, pCMV-VSV-G envelope, and pCMV-Gag/Pol packaging vectors. Supernatants containing the virus were collected 48 and 72 hours post-transfection, filtered through a 0.45 µm filter, and concentrated by ultracentrifugation at 25,000 rpm for 2 hours at 4°C through a 25% sucrose cushion [118]. The viral pellet was resuspended in RPMI medium and stored at -80°C. The virus stock titer was determined by infecting Jurkat cells with serial dilutions of viral-containing supernatants and detection of GFP expression by flow cytometry two days after cell transduction.

### Generation of latently infected Jurkat cells

Jurkat cells were infected with NL4.3Δ6-drGFP viral stock at an MOI of 0.2. Forty-eight hours post-infection, GFP+ (infected) cells were sorted using BD FACSAria II and the GFP+ population was maintained in culture and regularly monitored by flow cytometry for GFP expression until latent infection was established (2–3 weeks). For stimulation of latently infected Jurkat cells, 1 × 10⁵ cells were treated with the indicated concentrations of LRAs (S2 Table) for 24 hours, except for the FOXO-1 inhibitor, which was added for 48 hours. Cells were then analysed by flow cytometry to measure the percentage of GFP+ cells and the GFP MFI. AUY922,17-AAG and 5Z-7-Oxozeaenol (TAK1 inhibitor) were dissolved in DMSO and used at serial dilutions as indicated in the Figures. To assess the toxicity of the inhibitors, cells were stained with LIVE/DEAD Fixable blue dead cell stain (Thermo Fisher scientific) before analysis. For all Jurkat cells experiments, fluorescence was measured on a BD LSR Fortessa and analysed using the FlowJo software 10.8.1.

### Infection of primary CD4+ T cells and generation of latently infected cells

CD4+ T cells were activated by anti-CD3/CD28 monoclonal antibodies. Tissue culture plates were precoated with anti-CD3 (OKT) Ab at 1 µg/mL then cells were added to the plate together with soluble anti-CD28 Ab (CD28.2) at 2 µg/mL and 100 U/mL IL-2 in X-VIVO media supplemented with 5% FBS and 100 U/mL penicillin/streptomycin. On day 3, the activated CD4+ T cells were infected with the NL4.3Δ6-drGFP virus at an MOI of 2 in the presence of 4 µg/mL polybrene and 100 U/mL IL-2. The cells were incubated overnight on a shaker in the incubator. After removing them from the shaker, the cells were incubated for an additional 24 hours. An aliquot of the cells was then stained with CD3, CD4, CD45RA, CD45RO, CD25, and CD69 and analysed by flow cytometry to assess infection by measuring GFP+ cells within the CD45RO+ population. The cells were subsequently monitored for latency establishment by measuring GFP+ cells every other day. Once latency was established, the cells were reactivated using different LRAs. To reactivate latently infected CD4+ T cells, 1 × 10⁵ cells were cultured in 96-well plates in 100µL media and treated with different LRAs, including anti-CD3/CD28 (1 µg/mL/2 µg/mL), IL-7 + IL-15 (20 ng/mL of each), AS1842856 (200 nM), TLR7 agonist (5 μg/mL), and TLR8 agonist (5 μg/mL). Cells were treated with each LRA in the presence of either DMSO, AUY922 25 nM or 50 nM and incubated for 48 hours before staining and analysis by flow cytometry. Additionally, the supernatant of these cells was collected for cytokine analysis.

### Cell surface staining for flow cytometry

For surface marker staining, the appropriate human-specific mAbs were chosen (Table 1). For Jurkat cells and TPR cells, fluorescence was measured using a BD LSR Fortessa BD FACS Diva9 software, while for infected CD4+ T cells, fluorescence was measured using a Cytek Aurora. Data were analysed using FlowJo version 10.8.1. The gating strategy used to identify T cells subsets is shown in S4 Fig. For cell staining, the antibodies were prepared by diluting them 1:100 in FACS buffer. The diluted antibodies were then added to the cells and incubated for 30 minutes at 4°C in the dark. Following incubation, the cells were washed twice with PBS, resuspended in 200 µL of FACS buffer combined with 200 µL of 4% paraformaldehyde (PFA), and subsequently analyzed by flow cytometry.

**Table 1:** Markers, fluorochromes and antibodies for flow cytometry.

Fluorochrome	Specificity (Marker)	Supplier (catalogue number)
BV510	TIGIT	BD (747842)
BV711	Tim-3	BD (747959)
BV786	CD197(CCR7)	BD (566758)
BV421	CD196(CCR6)	BD (562515)
BV605	CD71	BD (745096)
APC/fire 810	CD25	BioLegend (356150)
APC	CD69	Biolegend (310910)
Alexa fluor 700	CD 127	BioLegend (351344)
APC/Cyanine 7	CD4+5RO	BioLegend (304228)
BUV805	HLA-DR	BD (748338)
UV (450) live dead blue	Live/Dead	ThermoFisher (L34961)
BUV 395	CD4+5RA	BD (740315)
BUV737	CD183	BD (741866)
Spark NIR 685	CD38	BioLegend (303552)
PE-Dazzle	PD-1	BioLegend (367434)
PE cy7	CD4+	Biolegend (317414)
PE/fire 810	CD194(CCR4)	BioLegend (359433)
PE	CTLA4(CD152)	BioLegend (369604)
GFP	GFP reporter	
Percp cy5.5	CD3	Biolegend (317336)

### Quantification of HIV-1 integration

To quantify the integration of HIV-1 in CD4+ T cells, nested Alu-LTR quantitative PCR was performed as previously described [114]. Briefly, DNA was isolated from infected CD4+ T cells 48 hours post-infection, 10 days post-infection, and from a negative sample (uninfected) using the Qiagen Blood Mini Kit. Integrated DNA was pre-amplified using 50 nM Alu forward primer, 150 nM HIV-1 LTR reverse primer, 25 μL PCR Master Mix (2X) (ThermoFisher), and 200 ng DNA in 50 μL reactions. Cycling conditions were: 95°C for 8 min x 1 cycle, followed by 18 cycles of 95°C for 1 min, 52°C for 1 min, and 72°C for 3 min. A second round real-time TaqMan quantitative PCR was performed using the pre-amplified DNA. These samples were run alongside a standard curve of known dilutions of infected Jurkat cells containing integrated HIV-1 DNA. Reactions contained 0.5 μM Alu forward primer, 0.5 μM Alu-LTR2 reverse primer, 0.15 μM probe, 10 μL 2x TaqMan Gene Expression Master Mix, and 2 μL of 1:20 diluted pre-amplified DNA. Cycling conditions were: 50°C for 2 min, 95°C for 15 min x 1 cycle, followed by 45 cycles of 95°C for 15 s, and 60°C for 1 min. Reactions were performed using a QuantStudio real-time PCR system. Below is the list of primers used:

ALU-FW: AAC TAG GGA ACC CAC TGC TTA AG
LTR1-RV: TGC TGG GAT TAC AGG CGT GAG
LTR2-RV: TGC TAG AGA TTT TCC ACA CTG ACT
ALU- Probe: FAM—TAG TGT GTG CCC GTC TGT TGT GTG AC—TAMRA

#### qPCR for proviral quantification.

Total DNA from 1.5 × 10^6^ infected WT or N74D Jurkat cells was extracted at each week after sorting using DNeasy blood & tissue kit (Qiagen). DNA concentration and purity were measured by Nonodrop and each DNA sample was normalized to 100ng/μl. The real-time qPCR reaction was performed using a Real-Time PCR machine (Applied Biosystems) in a final volume of 20 μl containing 1x Power-Up SYBR green master mix (Applied Biosystems), 200ng of DNA in 2 μl and 0.2 μM of each primer: forward GFP primer AAGCTGACCCTGAAGTTCATCTGC and reverse GFP primer CTTGTAGTTGCCGTGGTCCTTGAA. Cycling parameters were 95^0^C for 2 min followed by 95^0^C for 1 min, 55^0^C for 1 min and 68^0^C for 1 min repeated for 40 cycles. Quantification was done using a standard curve that was generated by serial dilutions of the Δ6-drEGFP plasmid.

### Western blot

Latently infected Jurkat cells were treated with the anti-CD3/CD28 Abs alone or in the presence of AUY922 for 24h. 1x10^6^ cells were collected and washed with cold PBS then incubated with 1X RIPA buffer and a protease inhibitor cocktail for 30 minutes on ice. Samples were centrifuged for 20 minutes at 4 °C ≥8000 x g and the cell lysate was stored at -20 °C. Cell lysate were diluted in 6X SDS buffer and 12 μl of cell lysate was loaded onto a Mini-PROTEAN TGX Stain-Free Precast gel (4–20%) (Bio-Rad). Samples were run for 1 hour at 120V, the proteins were transferred to a 0.2 μm Nitrocellulose membrane (Bio-Rad) at 25V, 2.5A, for 7 minutes using Trans-Blot Turbo system. The membrane was blocked for 2 hours with 5% skim milk in TBST (150 mM NaCl, 20 mM Tris base, 0.1% Tween 20), and incubated overnight at 4°C with the primary antibodies against TAK1, or alpha-actin diluted in 1% skim milk in TBST. The next day, the membrane was washed with TBST 3 times for 10 mins each before incubation with the HRP-conjugated secondary antibody for 1 hour at room temperature. The membrane was washed in TBST 3 times for 10 mins each time and the signal was detected using Chemiluminescence (ECL, Thermo Scientific) following the manufacturer’s instructions. Images were collected by ChemiDoc Imaging system (Bio-Rad) and analyzed by the Image Lab program.

### Cytokine analysis

Supernatants from CD4+ T cells isolated from five different donors were collected on the day of cells analysis. These supernatants were then analysed for IFN-γ, IL-4, IL-17A, IL-10, and TNF-α levels using the LEGENDplex Human Essential Immune Response Panel Mix and Match (Cat #740932, BioLegend) following the manufacturer’s protocol. The measurements were taken using a BD LSRFortessa flow cytometer, and the data were analysed with the LEGENDplex Data Analysis Software.

### Statistical analysis

Means ± SE or SD, n, and the statistical test used are shown in the Figure legends. Statistical analyses were conducted using GraphPad Prism software. The levels of statistical significance are indicated as follows: *p ≤ 0.05; **p ≤ 0.01; ***p ≤ 0.001; ****p < 0.0001.

## Supporting information

S1 Fig Optimisation of LRAs to reactivate HIV-1 in latently infected Jurkat cells. Latently infected Jurkat cells were activated for 24 hours (48 hours for the FOXO-1 inhibitor) with five different concentrations of (A) PMA, (B) PHA, (C) Ionomycin, (D) TLR7 or TLR8 agonists and (E) the FOXO-1 inhibitor. The cells were then analysed by flow cytometry to measure, from left to right, the percentage of GFP+ cells, GFP MFI, the percentage of CD69+ cells and cell viability using the same gating strategy shown in Fig 1E. Bar graphs show the average values ± SD (n = 6 for GFP) and (n = 3 for viability, CD69). Significance was calculated using a one-way ANOVA with Dunnett’s correction. *=p≤0.05; **=p≤0.01; ***=p≤0.001; ****=p<0.0001.
(TIF)

S2 Fig Optimisation of cytokines to reactivate HIV-1 in latently infected Jurkat cells. (A) Latently infected Jurkat cells were activated for 24 hours with different concentrations of TNF-α and analysed by flow cytometry to measure, from left to right, the percentage of GFP+ cells, GFP MFI, the percentage of CD69+ cells and cell viability using the same gating strategy shown in Fig 1E, n=6 for GFP and n = 3 for viability and CD69. (B) latently infected cells were stimulated with different concentrations of IL-2, IL-6, IL-7+IL-15, IL-7 alone or IL-15 alone and analysed by flow cytometry to measure the percentage of GFP+ cells and (C) the GFP MFI. Bar graphs show the average values ± SD, n = 3. (D) Cell viability was analysed by flow cytometry using forward vs. side-scatter profiles. (E) Jurkat cells and primary CD4+ T cells were stained for CD127, the IL-7 receptor. A representative flow cytometry plot shows the percentage of CD127+ cells in CD4+ T cells.
(TIF)

S3 Fig AUY922 represses HIV-1 reactivation in latently infected Jurkat cells with no detectable loss of cell viability. Latently infected Jurkat cells were treated with a fixed concentration of LRAs alone or with different concentrations of AUY922 for 24 hours, except for FOXO-1, which was incubated for 48 hours. Cells were then stained with live or dead blue stain and analysed by flow cytometry to assess cell viability.
(TIF)

S4 Fig 17AAG represses HIV-1 reactivation in latently infected Jurkat cells with no detectable loss of cell viability. Latently Jurkat cells were treated with a fixed concentration of LRAs (anti-CD3/CD28 Abs or PMA in panel A; PHA, ionomycin, TNF-α, FOXO-1 inhibitor, TLR7 or 8 agonists in panel B) alone or with different concentrations of 17AAG for 24 hours, except for FOXO-1, which was incubated for 48 hours. Cells were then stained with live or dead blue stain and analysed by flow cytometry to assess cell toxicity.
(TIF)

S5 Fig TPR cells activation by ionomycin. TPR cells were stimulated with different concentrations of ionomycin as indicated. (A) MFI was measured by flow cytometry to detect activation of NF-kB (left panel), NFAT (middle panel) and AP-1 (right panel). (B) Graph showing average MFI values ± SD for each transcription factor, (n= 3).
(TIF)

S6 Fig TPR cells activation with anti-CD3/CD28 antibodies. (A) TPR cells expressing a reconstituted TCR were stimulated with anti CD3 [1μg/ml]/ anti CD28 [2μg/ml] antibodies in the presence of the indicated concentrations of AUY922. MFI was measured by flow cytometry to detect activation of NF-kB (left panel), NFAT (middle panel) and AP-1 (right panel). (B) Graph showing average MFI values ± SD for each transcription factor (n= 3). (C) The same TPR cells were stimulated with anti CD3 [1μg/ml]/ anti CD28 [2μg/ml] antibodies in the presence of the indicated concentrations of 5Z. MFI was measured by flow cytometry to detect activation of NF-kB (left panel), NFAT (middle panel) and AP-1 (right panel). (D) Graph showing average MFI values ± SD for each transcription factor (n= 3).
(TIF)

S7 Fig Gating strategy and FMO. (A) Primary CD4+ T cells were analysed by spectral flow cytometry and positive gates were established by staining with the 18-antibody panel minus one (fluorescence minus one or FMO). (B) Representative flow cytometry plots and gating strategy of T cell subsets from one donor gated from live CD3+CD4+ cells.
(TIF)

S8 Fig The effect of AUY922 treatment on different CD4+ T cell populations. CD4+ T cells were isolated from PBMCs and treated with IL-2 only (No stim) or anti-CD3/CD28 Abs + IL-2 for 72 hours, and AUY922 [25 nM] or DMSO added 48 hours post stimulation. Cells were analysed by flow cytometry 24 hours after the addition of AUY922. tSNE data of the T subsets, activation, and inhibitory markers were generated by FlowJo. A) tSNE plot for donor 2. B) tSNE plot for donor 3. C) tSNE plot for donor 4.
(TIF)

S9 Fig Gating strategy for GFP+ cells in T cell subsets. A) Representative flow cytometry plots of Live/dead gating. (B-D) Representative flow cytometry plots of GFP+ cells in T cell subsets from one donor gated from live CD3+CD4+ cells after stimulation with anti-CD3/CD28 antibodies (B), IL7/IL15 (C), or FOXO-1 inhibitor (D).
(TIF)

S10 Fig Effect of AUY922 on latently infected primary CD4+ T cells treated with IL-2 only. Latently infected primary CD4+ T cells were generated ex vivo as described in Fig 7A. On day 9 or 11, cells were treated with AUY922 [25 nM or 50 nM] or DMSO in the presence of IL-2. Graphs showing the results for 2 donors: GFP MFI (left panel), percentage of GFP+ cells (middle panel), and combined % GFP x MFI in the CD45RA- CD45RO+ population.
(TIF)

S11 Fig The effect of AUY92 on cytokines production. Supernatant from latently infected primary CD4+ T cells, which were re-stimulated with anti-CD3/CD28 Abs in the presence or absence of DMSO, AUY922 (25 nM), or AUY922 (50 nM) was collected 48 hours after re-stimulation and used to measure different cytokines concentration. (A) IFN-γ, (B) IL-4, (C) IL-17A, (D) IL-10, and (E) TNF-α. Data are presented as mean ± standard error of the mean (SEM) n=5. Concentrations are shown in pg/ml. Statistical significance was determined using a Paired two-tailed Student’s t-test. *P < 0.05, **P < 0.01, ***P < 0.001. Pairwise comparisons were: No stim-IL-2 and anti-CD3/CD28; anti-CD3/CD28 and AUY922 25nM; anti-CD3/CD28 and AUY922 50 nM.
(TIF)

S1 Table LRAs used to reactivate HIV-1 in latently infected Jurkat cells. (XLSX)

S2 Table Concentrations of LRAs used to reactivate latent HIV-1 in Jurkat T cells. (XLSX)

S3 Table T cell markers used for flow cytometry. (XLSX)

S4 Table Markers used to identify T cell subsets, activation and inhibition. (XLSX)

## References

[1] Simon V, Ho DD, Abdool Karim Q. HIV/AIDS epidemiology, pathogenesis, prevention, and treatment. Lancet. 2006;368(9534):489–504. doi: 10.1016/S0140-6736(06)69157-5 16890836 PMC2913538

[2] Heaton RK, Clifford DB, Franklin DR Jr, Woods SP, Ake C, Vaida F, et al. HIV-associated neurocognitive disorders persist in the era of potent antiretroviral therapy: CHARTER Study. Neurology. 2010;75(23):2087–96. doi: 10.1212/WNL.0b013e318200d727 21135382 PMC2995535

[3] Trickey A, Sabin CA, Burkholder G, Crane H, d’Arminio Monforte A, Egger M, et al. Life expectancy after 2015 of adults with HIV on long-term antiretroviral therapy in Europe and North America: a collaborative analysis of cohort studies. Lancet HIV. 2023;10(5):e295–307. doi: 10.1016/S2352-3018(23)00028-0 36958365 PMC10288029

[4] Colby DJ, Trautmann L, Pinyakorn S, Leyre L, Pagliuzza A, Kroon E, et al. Rapid HIV RNA rebound after antiretroviral treatment interruption in persons durably suppressed in Fiebig I acute HIV infection. Nat Med. 2018;24(7):923–6. doi: 10.1038/s41591-018-0026-6 29892063 PMC6092240

[5] Cohn LB, Chomont N, Deeks SG. The biology of the HIV-1 latent reservoir and implications for cure strategies. Cell Host Microbe. 2020;27(4):519–30. doi: 10.1016/j.chom.2020.03.014 32272077 PMC7219958

[6] Veenhuis RT, Abreu CM, Shirk EN, Gama L, Clements JE. HIV replication and latency in monocytes and macrophages. Semin Immunol. 2021;51:101472. doi: 10.1016/j.smim.2021.101472 33648815 PMC10171083

[7] Armani-Tourret M, Bone B, Tan TS, Sun W, Bellefroid M, Struyve T, et al. Immune targeting of HIV-1 reservoir cells: a path to elimination strategies and cure. Nat Rev Microbiol. 2024;22(6):328–44. doi: 10.1038/s41579-024-01010-8 38337034 PMC11131351

[8] Utay NS, Hunt PW. Role of immune activation in progression to AIDS. Curr Opin HIV AIDS. 2016;11(2):131–7. doi: 10.1097/COH.0000000000000242 26731430 PMC4750472

[9] Schouten J, Wit FW, Stolte IG, Kootstra NA, van der Valk M, Geerlings SE, et al. Cross-sectional comparison of the prevalence of age-associated comorbidities and their risk factors between HIV-infected and uninfected individuals: the AGEhIV cohort study. Clin Infect Dis. 2014;59(12):1787–97. doi: 10.1093/cid/ciu701 25182245

[10] Buck AM, LaFranchi BH, Henrich TJ. Gaining momentum: stem cell therapies for HIV cure. Curr Opin HIV AIDS. 2024;19(4):194–200. doi: 10.1097/COH.0000000000000859 38686850 PMC11155292

[11] Siliciano JD, Siliciano RF. HIV cure: the daunting scale of the problem. Science. 2024;383(6684):703–5. doi: 10.1126/science.adk1831 38359111

[12] Pereira LA, Bentley K, Peeters A, Churchill MJ, Deacon NJ. A compilation of cellular transcription factor interactions with the HIV-1 LTR promoter. Nucleic Acids Res. 2000;28(3):663–8. doi: 10.1093/nar/28.3.663 10637316 PMC102541

[13] Anderson I, Low JS, Weston S, Weinberger M, Zhyvoloup A, Labokha AA, et al. Heat shock protein 90 controls HIV-1 reactivation from latency. Proc Natl Acad Sci U S A. 2014;111(15):E1528-37. doi: 10.1073/pnas.1320178111 24706778 PMC3992654

[14] Bell B, Sadowski I. Ras-responsiveness of the HIV-1 LTR requires RBF-1 and RBF-2 binding sites. Oncogene. 1996;13(12):2687–97. 9000143

[15] Brooks DG, Arlen PA, Gao L, Kitchen CMR, Zack JA. Identification of T cell-signaling pathways that stimulate latent HIV in primary cells. Proc Natl Acad Sci U S A. 2003;100(22):12955–60. doi: 10.1073/pnas.2233345100 14569007 PMC240726

[16] Chun TW, Engel D, Mizell SB, Ehler LA, Fauci AS. Induction of HIV-1 replication in latently infected CD4+ T cells using a combination of cytokines. J Exp Med. 1998;188(1):83–91. doi: 10.1084/jem.188.1.83 9653086 PMC2525548

[17] Dobrowolski C, Valadkhan S, Graham AC, Shukla M, Ciuffi A, Telenti A, et al. Entry of polarized effector cells into quiescence forces HIV latency. mBio. 2019;10(2):e00337-19. doi: 10.1128/mBio.00337-19 30914509 PMC6437053

[18] Van Lint C, Bouchat S, Marcello A. HIV-1 transcription and latency: an update. Retrovirology. 2013;10:67. doi: 10.1186/1742-4690-10-67 23803414 PMC3699421

[19] Mbonye U, Karn J. The cell biology of HIV-1 latency and rebound. Retrovirology. 2024;21(1):6. doi: 10.1186/s12977-024-00639-w 38580979 PMC10996279

[20] Mbonye U, Leskov K, Shukla M, Valadkhan S, Karn J. Biogenesis of P-TEFb in CD4+ T cells to reverse HIV latency is mediated by protein kinase C (PKC)-independent signaling pathways. PLoS Pathog. 2021;17(9):e1009581. doi: 10.1371/journal.ppat.1009581 34529720 PMC8478230

[21] Chatterjee D, Zhang Y, Ngassaki-Yoka CD, Dutilleul A, Khalfi S, Hernalsteens O, et al. Identification of aryl hydrocarbon receptor as a barrier to HIV-1 infection and outgrowth in CD4(+) T cells. Cell Rep. 2023;42(6):112634. doi: 10.1016/j.celrep.2023.112634 37310858 PMC10592455

[22] Wiche Salinas TR, Zhang Y, Sarnello D, Zhyvoloup A, Marchand LR, Fert A, et al. Th17 cell master transcription factor RORC2 regulates HIV-1 gene expression and viral outgrowth. Proc Natl Acad Sci U S A. 2021;118(48):e2105927118. doi: 10.1073/pnas.2105927118 34819367 PMC8640723

[23] Li C, Mori L, Valente ST. The block-and-lock strategy for human immunodeficiency virus cure: lessons learned from didehydro-cortistatin A. J Infect Dis. 2021;223(12 Suppl 2):46–53. doi: 10.1093/infdis/jiaa681 33586776 PMC7883021

[24] Li C, Mousseau G, Valente ST. Tat inhibition by didehydro-cortistatin A promotes heterochromatin formation at the HIV-1 long terminal repeat. Epigenetics Chromatin. 2019;12(1):23. doi: 10.1186/s13072-019-0267-8 30992052 PMC6466689

[25] Mediouni S, Chinthalapudi K, Ekka MK, Usui I, Jablonski JA, Clementz MA, et al. Didehydro-Cortistatin A inhibits HIV-1 by specifically binding to the unstructured basic region of tat. mBio. 2019;10(1):e02662-18. doi: 10.1128/mBio.02662-18 30723126 PMC6368365

[26] Vozzolo L, Loh B, Gane PJ, Tribak M, Zhou L, Anderson I, et al. Gyrase B inhibitor impairs HIV-1 replication by targeting Hsp90 and the capsid protein. J Biol Chem. 2010;285(50):39314–28. doi: 10.1074/jbc.M110.155275 20937817 PMC2998086

[27] Roesch F, Meziane O, Kula A, Nisole S, Porrot F, Anderson I, et al. Hyperthermia stimulates HIV-1 replication. PLoS Pathog. 2012;8(7):e1002792. doi: 10.1371/journal.ppat.1002792 22807676 PMC3395604

[28] Joshi P, Maidji E, Stoddart CA. Inhibition of heat shock protein 90 prevents HIV rebound. J Biol Chem. 2016;291(19):10332–46. doi: 10.1074/jbc.M116.717538 26957545 PMC4858980

[29] Pan X-Y, Zhao W, Zeng X-Y, Lin J, Li M-M, Shen X-T, et al. Heat shock factor 1 mediates latent HIV reactivation. Sci Rep. 2016;6:26294. doi: 10.1038/srep26294 27189267 PMC4870680

[30] Low JS, Fassati A. Hsp90: a chaperone for HIV-1. Parasitology. 2014;141(9):1192–202. doi: 10.1017/S0031182014000298 25004926

[31] Painter MM, Zaikos TD, Collins KL. Quiescence promotes latent HIV infection and resistance to reactivation from latency with histone deacetylase inhibitors. J Virol. 2017;91(24):e01080-17. doi: 10.1128/JVI.01080-17 29021396 PMC5709582

[32] Pan X-Y, Zhao W, Wang C-Y, Lin J, Zeng X-Y, Ren R-X, et al. Heat shock protein 90 facilitates latent HIV reactivation through maintaining the function of Positive Transcriptional Elongation Factor b (p-TEFb) under proteasome inhibition. J Biol Chem. 2016;291(50):26177–87. doi: 10.1074/jbc.M116.743906 27799305 PMC5207085

[33] Peng W, Hong Z, Chen X, Gao H, Dai Z, Zhao J, et al. Thiostrepton reactivates latent HIV-1 through the p-TEFb and NF-κB pathways mediated by heat shock response. Antimicrob Agents Chemother. 2020;64(5):e02328-19. doi: 10.1128/AAC.02328-19 32094131 PMC7179580

[34] O’Keeffe B, Fong Y, Chen D, Zhou S, Zhou Q. Requirement for a kinase-specific chaperone pathway in the production of a Cdk9/cyclin T1 heterodimer responsible for P-TEFb-mediated tat stimulation of HIV-1 transcription. J Biol Chem. 2000;275(1):279–87. doi: 10.1074/jbc.275.1.279 10617616

[35] Li Z-N, Luo Y. HSP90 inhibitors and cancer: prospects for use in targeted therapies (Review). Oncol Rep. 2023;49(1):6. doi: 10.3892/or.2022.8443 36367182 PMC9685368

[36] Sessa C, Shapiro GI, Bhalla KN, Britten C, Jacks KS, Mita M, et al. First-in-human phase I dose-escalation study of the HSP90 inhibitor AUY922 in patients with advanced solid tumors. Clin Cancer Res. 2013;19(13):3671–80. doi: 10.1158/1078-0432.CCR-12-3404 23757357

[37] Taipale M, Jarosz DF, Lindquist S. HSP90 at the hub of protein homeostasis: emerging mechanistic insights. Nat Rev Mol Cell Biol. 2010;11(7):515–28. doi: 10.1038/nrm2918 20531426

[38] Boulon S, Pradet-Balade B, Verheggen C, Molle D, Boireau S, Georgieva M, et al. HSP90 and its R2TP/Prefoldin-like cochaperone are involved in the cytoplasmic assembly of RNA polymerase II. Mol Cell. 2010;39(6):912–24. doi: 10.1016/j.molcel.2010.08.023 20864038 PMC4333224

[39] Jordan A, Bisgrove D, Verdin E. HIV reproducibly establishes a latent infection after acute infection of T cells in vitro. EMBO J. 2003;22(8):1868–77. doi: 10.1093/emboj/cdg188 12682019 PMC154479

[40] Spina CA, Anderson J, Archin NM, Bosque A, Chan J, Famiglietti M, et al. An in-depth comparison of latent HIV-1 reactivation in multiple cell model systems and resting CD4+ T cells from aviremic patients. PLoS Pathog. 2013;9(12):e1003834. doi: 10.1371/journal.ppat.1003834 24385908 PMC3873446

[41] Saleh S, Wightman F, Ramanayake S, Alexander M, Kumar N, Khoury G, et al. Expression and reactivation of HIV in a chemokine induced model of HIV latency in primary resting CD4+ T cells. Retrovirology. 2011;8:80. doi: 10.1186/1742-4690-8-80 21992606 PMC3215964

[42] Covino DA, Desimio MG, Doria M. Impact of IL-15 and latency reversing agent combinations in the reactivation and NK cell-mediated suppression of the HIV reservoir. Sci Rep. 2022;12(1):18567. doi: 10.1038/s41598-022-23010-5 36329160 PMC9633760

[43] Chen H-C, Martinez JP, Zorita E, Meyerhans A, Filion GJ. Position effects influence HIV latency reversal. Nat Struct Mol Biol. 2017;24(1):47–54. doi: 10.1038/nsmb.3328 27870832

[44] Vansant G, Chen H-C, Zorita E, Trejbalová K, Miklík D, Filion G, et al. The chromatin landscape at the HIV-1 provirus integration site determines viral expression. Nucleic Acids Res. 2020;48(14):7801–17. doi: 10.1093/nar/gkaa536 32597987 PMC7641320

[45] Zhyvoloup A, Melamed A, Anderson I, Planas D, Lee C-H, Kriston-Vizi J, et al. Digoxin reveals a functional connection between HIV-1 integration preference and T-cell activation. PLoS Pathog. 2017;13(7):e1006460. doi: 10.1371/journal.ppat.1006460 28727807 PMC5519191

[46] Yang H-C, Xing S, Shan L, O’Connell K, Dinoso J, Shen A, et al. Small-molecule screening using a human primary cell model of HIV latency identifies compounds that reverse latency without cellular activation. J Clin Invest. 2009;119(11):3473–86. doi: 10.1172/JCI39199 19805909 PMC2769176

[47] Bosque A, Planelles V. Induction of HIV-1 latency and reactivation in primary memory CD4+ T cells. Blood. 2009;113(1):58–65. doi: 10.1182/blood-2008-07-168393 18849485 PMC2614643

[48] Meås HZ, Haug M, Beckwith MS, Louet C, Ryan L, Hu Z, et al. Sensing of HIV-1 by TLR8 activates human T cells and reverses latency. Nat Commun. 2020;11(1):147. doi: 10.1038/s41467-019-13837-4 31919342 PMC6952430

[49] Simms PE, Ellis TM. Utility of flow cytometric detection of CD69 expression as a rapid method for determining poly- and oligoclonal lymphocyte activation. Clin Diagn Lab Immunol. 1996;3(3):301–4. doi: 10.1128/cdli.3.3.301-304.1996 8705673 PMC170336

[50] Tsai A, Irrinki A, Kaur J, Cihlar T, Kukolj G, Sloan DD, et al. Toll-like receptor 7 agonist GS-9620 induces HIV expression and HIV-specific immunity in cells from HIV-infected individuals on suppressive antiretroviral therapy. J Virol. 2017;91(8):e02166-16. doi: 10.1128/JVI.02166-16 28179531 PMC5375698

[51] Vallejo-Gracia A, Chen IP, Perrone R, Besnard E, Boehm D, Battivelli E, et al. FOXO1 promotes HIV latency by suppressing ER stress in T cells. Nat Microbiol. 2020;5(9):1144–57. doi: 10.1038/s41564-020-0742-9 32541947 PMC7483895

[52] Roux A, Leroy H, De Muylder B, Bracq L, Oussous S, Dusanter-Fourt I, et al. FOXO1 transcription factor plays a key role in T cell-HIV-1 interaction. PLoS Pathog. 2019;15(5):e1007669. doi: 10.1371/journal.ppat.1007669 31042779 PMC6513100

[53] Eccles SA, Massey A, Raynaud FI, Sharp SY, Box G, Valenti M, et al. NVP-AUY922: a novel heat shock protein 90 inhibitor active against xenograft tumor growth, angiogenesis, and metastasis. Cancer Res. 2008;68(8):2850–60. doi: 10.1158/0008-5472.CAN-07-5256 18413753

[54] Neckers L, Workman P. Hsp90 molecular chaperone inhibitors: are we there yet? Clin Cancer Res. 2012;18(1):64–76. doi: 10.1158/1078-0432.CCR-11-1000 22215907 PMC3252205

[55] Brough PA, Aherne W, Barril X, Borgognoni J, Boxall K, Cansfield JE, et al. 4,5-diarylisoxazole Hsp90 chaperone inhibitors: potential therapeutic agents for the treatment of cancer. J Med Chem. 2008;51(2):196–218. doi: 10.1021/jm701018h 18020435

[56] Battivelli E, Dahabieh MS, Abdel-Mohsen M, Svensson JP, Tojal Da Silva I, Cohn LB, et al. Distinct chromatin functional states correlate with HIV latency reactivation in infected primary CD4+ T cells. Elife. 2018;7:e34655. doi: 10.7554/eLife.34655 29714165 PMC5973828

[57] Dar RD, Hosmane NN, Arkin MR, Siliciano RF, Weinberger LS. Screening for noise in gene expression identifies drug synergies. Science. 2014;344(6190):1392–6. doi: 10.1126/science.1250220 24903562 PMC4122234

[58] Schulte TW, Neckers LM. The benzoquinone ansamycin 17-allylamino-17-demethoxygeldanamycin binds to HSP90 and shares important biologic activities with geldanamycin. Cancer Chemother Pharmacol. 1998;42(4):273–9. doi: 10.1007/s002800050817 9744771

[59] Hwang J-R, Byeon Y, Kim D, Park S-G. Recent insights of T cell receptor-mediated signaling pathways for T cell activation and development. Exp Mol Med. 2020;52(5):750–61. doi: 10.1038/s12276-020-0435-8 32439954 PMC7272404

[60] Shah K, Al-Haidari A, Sun J, Kazi JU. T cell receptor (TCR) signaling in health and disease. Signal Transduct Target Ther. 2021;6(1):412. doi: 10.1038/s41392-021-00823-w 34897277 PMC8666445

[61] Lee J, Mira-Arbibe L, Ulevitch RJ. TAK1 regulates multiple protein kinase cascades activated by bacterial lipopolysaccharide. J Leukoc Biol. 2000;68(6):909–15. doi: 10.1189/jlb.68.6.909 11129660

[62] Ninomiya-Tsuji J, Kishimoto K, Hiyama A, Inoue J, Cao Z, Matsumoto K. The kinase TAK1 can activate the NIK-I kappaB as well as the MAP kinase cascade in the IL-1 signalling pathway. Nature. 1999;398(6724):252–6. doi: 10.1038/18465 10094049

[63] Shim J-H, Xiao C, Paschal AE, Bailey ST, Rao P, Hayden MS, et al. TAK1, but not TAB1 or TAB2, plays an essential role in multiple signaling pathways in vivo. Genes Dev. 2005;19(22):2668–81. doi: 10.1101/gad.1360605 16260493 PMC1283960

[64] Wang X-D, Zhao C-S, Wang Q-L, Zeng Q, Feng X-Z, Li L, et al. The p38-interacting protein p38IP suppresses TCR and LPS signaling by targeting TAK1. EMBO Rep. 2020;21(7):e48035. doi: 10.15252/embr.201948035 32410369 PMC7332986

[65] Sun L, Deng L, Ea C-K, Xia Z-P, Chen ZJ. The TRAF6 ubiquitin ligase and TAK1 kinase mediate IKK activation by BCL10 and MALT1 in T lymphocytes. Mol Cell. 2004;14(3):289–301. doi: 10.1016/s1097-2765(04)00236-9 15125833

[66] Zhu L, Lama S, Tu L, Dusting GJ, Wang J-H, Liu G-S. TAK1 signaling is a potential therapeutic target for pathological angiogenesis. Angiogenesis. 2021;24(3):453–70. doi: 10.1007/s10456-021-09787-5 33973075

[67] Shi L, Zhang Z, Fang S, Xu J, Liu J, Shen J, et al. Heat shock protein 90 (Hsp90) regulates the stability of transforming growth factor beta-activated kinase 1 (TAK1) in interleukin-1beta-induced cell signaling. Mol Immunol. 2009;46(4):541–50. doi: 10.1016/j.molimm.2008.07.019 18950863

[68] Liu XY, Seh CC, Cheung PCF. HSP90 is required for TAK1 stability but not for its activation in the pro-inflammatory signaling pathway. FEBS Lett. 2008;582(29):4023–31. doi: 10.1016/j.febslet.2008.10.053 19026643

[69] Liu R, Lin Y, Jia R, Geng Y, Liang C, Tan J, et al. HIV-1 Vpr stimulates NF-κB and AP-1 signaling by activating TAK1. Retrovirology. 2014;11:45. doi: 10.1186/1742-4690-11-45 24912525 PMC4057933

[70] Wu J, Powell F, Larsen NA, Lai Z, Byth KF, Read J, et al. Mechanism and in vitro pharmacology of TAK1 inhibition by (5Z)-7-Oxozeaenol. ACS Chem Biol. 2013;8(3):643–50. doi: 10.1021/cb3005897 23272696

[71] Jutz S, Leitner J, Schmetterer K, Doel-Perez I, Majdic O, Grabmeier-Pfistershammer K, et al. Assessment of costimulation and coinhibition in a triple parameter T cell reporter line: Simultaneous measurement of NF-κB, NFAT and AP-1. J Immunol Methods. 2016;430:10–20. doi: 10.1016/j.jim.2016.01.007 26780292

[72] Liu Q, Busby JC, Molkentin JD. Interaction between TAK1-TAB1-TAB2 and RCAN1-calcineurin defines a signalling nodal control point. Nat Cell Biol. 2009;11(2):154–61. doi: 10.1038/ncb1823 19136967 PMC2656285

[73] Mousset CM, Hobo W, Woestenenk R, Preijers F, Dolstra H, van der Waart AB. Comprehensive phenotyping of T cells using flow cytometry. Cytometry A. 2019;95(6):647–54. doi: 10.1002/cyto.a.23724 30714682

[74] van den Broek T, Borghans JAM, van Wijk F. The full spectrum of human naive T cells. Nat Rev Immunol. 2018;18(6):363–73. doi: 10.1038/s41577-018-0001-y 29520044

[75] Kumar BV, Connors TJ, Farber DL. Human T cell development, localization, and function throughout life. Immunity. 2018;48(2):202–13. doi: 10.1016/j.immuni.2018.01.007 29466753 PMC5826622

[76] Jameson SC, Masopust D. Understanding subset diversity in T cell memory. Immunity. 2018;48(2):214–26. doi: 10.1016/j.immuni.2018.02.010 29466754 PMC5863745

[77] Golubovskaya V, Wu L. Different subsets of T cells, memory, effector functions, and CAR-T immunotherapy. Cancers (Basel). 2016;8(3):36. doi: 10.3390/cancers8030036 26999211 PMC4810120

[78] Raphael I, Nalawade S, Eagar TN, Forsthuber TG. T cell subsets and their signature cytokines in autoimmune and inflammatory diseases. Cytokine. 2015;74(1):5–17. doi: 10.1016/j.cyto.2014.09.011 25458968 PMC4416069

[79] Maecker HT, McCoy JP, Nussenblatt R. Standardizing immunophenotyping for the human immunology project. Nat Rev Immunol. 2012;12(3):191–200. doi: 10.1038/nri3158 22343568 PMC3409649

[80] Aspalter RM, Eibl MM, Wolf HM. Regulation of TCR-mediated T cell activation by TNF-RII. J Leukoc Biol. 2003;74(4):572–82. doi: 10.1189/jlb.0303112 12960285

[81] Fenwick C, Joo V, Jacquier P, Noto A, Banga R, Perreau M, et al. T-cell exhaustion in HIV infection. Immunol Rev. 2019;292(1):149–63. doi: 10.1111/imr.12823 31883174 PMC7003858

[82] Pardons M, Baxter AE, Massanella M, Pagliuzza A, Fromentin R, Dufour C, et al. Single-cell characterization and quantification of translation-competent viral reservoirs in treated and untreated HIV infection. PLoS Pathog. 2019;15(2):e1007619. doi: 10.1371/journal.ppat.1007619 30811499 PMC6411230

[83] Xie G, Luo X, Ma T, Frouard J, Neidleman J, Hoh R, et al. Characterization of HIV-induced remodeling reveals differences in infection susceptibility of memory CD4(+) T cell subsets in vivo. Cell Rep. 2021;35(4):109038. doi: 10.1016/j.celrep.2021.109038 ; PMCID: PMC8202093. 33910003 PMC8202093

[84] Van der Maaten L, Hinton G. Visualizing data using t-SNE. J Mach Learn Res. 2008;9(11).

[85] Zhou L, Sokolskaja E, Jolly C, James W, Cowley SA, Fassati A. Transportin 3 promotes a nuclear maturation step required for efficient HIV-1 integration. PLoS Pathog. 2011;7(8):e1002194. doi: 10.1371/journal.ppat.1002194 21901095 PMC3161976

[86] Geginat J, Lanzavecchia A, Sallusto F. Proliferation and differentiation potential of human CD8+ memory T-cell subsets in response to antigen or homeostatic cytokines. Blood. 2003;101(11):4260–6. doi: 10.1182/blood-2002-11-3577 12576317

[87] Shipkova M, Wieland E. Surface markers of lymphocyte activation and markers of cell proliferation. Clin Chim Acta. 2012;413(17–18):1338–49. doi: 10.1016/j.cca.2011.11.006 22120733

[88] Farber DL, Yudanin NA, Restifo NP. Human memory T cells: generation, compartmentalization and homeostasis. Nat Rev Immunol. 2014;14(1):24–35. doi: 10.1038/nri3567 24336101 PMC4032067

[89] Mbonye U, Wang B, Gokulrangan G, Shi W, Yang S, Karn J. Cyclin-dependent kinase 7 (CDK7)-mediated phosphorylation of the CDK9 activation loop promotes P-TEFb assembly with Tat and proviral HIV reactivation. J Biol Chem. 2018;293(26):10009–25. doi: 10.1074/jbc.RA117.001347 29743242 PMC6028954

[90] Kim YS, Alarcon SV, Lee S, Lee M-J, Giaccone G, Neckers L, et al. Update on Hsp90 inhibitors in clinical trial. Curr Top Med Chem. 2009;9(15):1479–92. doi: 10.2174/156802609789895728 19860730 PMC7241864

[91] Schnaider T, Somogyi J, Csermely P, Szamel M. The Hsp90-specific inhibitor geldanamycin selectively disrupts kinase-mediated signaling events of T-lymphocyte activation. Cell Stress Chaperones. 2000;5(1):52–61. doi: 10.1043/1355-8145(2000)005<0052:THSIGS>2.0.CO;2 10701840 PMC312910

[92] Hayashi K, Kamikawa Y. HSP90 is crucial for regulation of LAT expression in activated T cells. Mol Immunol. 2011;48(6–7):941–6. doi: 10.1016/j.molimm.2010.12.014 21251717

[93] Smith-Garvin JE, Koretzky GA, Jordan MS. T cell activation. Annu Rev Immunol. 2009;27:591–619. doi: 10.1146/annurev.immunol.021908.132706 19132916 PMC2740335

[94] Bennett LD, Fox JM, Signoret N. Mechanisms regulating chemokine receptor activity. Immunology. 2011;134(3):246–56. doi: 10.1111/j.1365-2567.2011.03485.x 21977995 PMC3209565

[95] Sallusto F, Kremmer E, Palermo B, Hoy A, Ponath P, Qin S, et al. Switch in chemokine receptor expression upon TCR stimulation reveals novel homing potential for recently activated T cells. Eur J Immunol. 1999;29(6):2037–45. doi: 10.1002/(SICI)1521-4141(199906)29:06<2037::AID-IMMU2037>3.0.CO;2-V 10382767

[96] Chiosis G, Neckers L. Tumor selectivity of Hsp90 inhibitors: the explanation remains elusive. ACS Chem Biol. 2006;1(5):279–84. doi: 10.1021/cb600224w 17163756

[97] Kamal A, Thao L, Sensintaffar J, Zhang L, Boehm MF, Fritz LC, et al. A high-affinity conformation of Hsp90 confers tumour selectivity on Hsp90 inhibitors. Nature. 2003;425(6956):407–10. doi: 10.1038/nature01913 14508491

[98] Unutmaz D, KewalRamani VN, Marmon S, Littman DR. Cytokine signals are sufficient for HIV-1 infection of resting human T lymphocytes. J Exp Med. 1999;189(11):1735–46. doi: 10.1084/jem.189.11.1735 10359577 PMC2193071

[99] Ducrey-Rundquist O, Guyader M, Trono D. Modalities of interleukin-7-induced human immunodeficiency virus permissiveness in quiescent T lymphocytes. J Virol. 2002;76(18):9103–11. doi: 10.1128/jvi.76.18.9103-9111.2002 12186894 PMC136444

[100] Reuschl AK, Mesner D, Shivkumar M, Whelan MVX, Pallett LJ, Guerra-Assuncao JA, et al. HIV-1 Vpr drives a tissue residency-like phenotype during selective infection of resting memory T cells. Cell Rep. 2022;39(2):110650. doi: 10.1016/j.celrep.2022.110650 35417711 PMC9350556

[101] Scripture-Adams DD, Brooks DG, Korin YD, Zack JA. Interleukin-7 induces expression of latent human immunodeficiency virus type 1 with minimal effects on T-cell phenotype. J Virol. 2002;76(24):13077–82. doi: 10.1128/jvi.76.24.13077-13082.2002 12438635 PMC136703

[102] Wang F-X, Xu Y, Sullivan J, Souder E, Argyris EG, Acheampong EA, et al. IL-7 is a potent and proviral strain-specific inducer of latent HIV-1 cellular reservoirs of infected individuals on virally suppressive HAART. J Clin Invest. 2005;115(1):128–37. doi: 10.1172/JCI22574 15630452 PMC539197

[103] Mohammadi P, di Iulio J, Muñoz M, Martinez R, Bartha I, Cavassini M, et al. Dynamics of HIV latency and reactivation in a primary CD4+ T cell model. PLoS Pathog. 2014;10(5):e1004156. doi: 10.1371/journal.ppat.1004156 24875931 PMC4038609

[104] Bosque A, Famiglietti M, Weyrich AS, Goulston C, Planelles V. Homeostatic proliferation fails to efficiently reactivate HIV-1 latently infected central memory CD4+ T cells. PLoS Pathog. 2011;7(10):e1002288. doi: 10.1371/journal.ppat.1002288 21998586 PMC3188522

[105] Gattinoni L, Speiser DE, Lichterfeld M, Bonini C. T memory stem cells in health and disease. Nat Med. 2017;23(1):18–27. doi: 10.1038/nm.4241 28060797 PMC6354775

[106] Fromentin R, Chomont N, editors. HIV persistence in subsets of CD4+ T cells: 50 shades of reservoirs. Semin Immunol; 2021. 10.1016/j.smim.2020.101438 PMC8164644 33272901

[107] Kulpa DA, Talla A, Brehm JH, Ribeiro SP, Yuan S, Bebin-Blackwell A-G, et al. Differentiation into an effector memory phenotype potentiates HIV-1 latency reversal in CD4+ T cells. J Virol. 2019;93(24):e00969-19. doi: 10.1128/JVI.00969-19 31578289 PMC6880164

[108] Grau-Expósito J, Luque-Ballesteros L, Navarro J, Curran A, Burgos J, Ribera E, et al. Latency reversal agents affect differently the latent reservoir present in distinct CD4+ T subpopulations. PLoS Pathog. 2019;15(8):e1007991. doi: 10.1371/journal.ppat.1007991 31425551 PMC6715238

[109] Baxter AE, Niessl J, Fromentin R, Richard J, Porichis F, Charlebois R, et al. Single-cell characterization of viral translation-competent reservoirs in HIV-infected individuals. Cell Host Microbe. 2016;20(3):368–80. doi: 10.1016/j.chom.2016.07.015 27545045 PMC5025389

[110] Magyar CTJ, Vashist YK, Stroka D, Kim-Fuchs C, Berger MD, Banz VM. Heat shock protein 90 (HSP90) inhibitors in gastrointestinal cancer: where do we currently stand?-A systematic review. J Cancer Res Clin Oncol. 2023;149(10):8039–50. doi: 10.1007/s00432-023-04689-z 36966394 PMC10374781

[111] Lang JE, Forero-Torres A, Yee D, Yau C, Wolf D, Park J, et al. Safety and efficacy of HSP90 inhibitor ganetespib for neoadjuvant treatment of stage II/III breast cancer. NPJ Breast Cancer. 2022;8(1):128. doi: 10.1038/s41523-022-00493-z 36456573 PMC9715670

[112] Janssens J, Kim P, Kim SJ, Wedrychowski A, Kadiyala GN, Hunt PW, et al. Mechanisms and efficacy of small molecule latency-promoting agents to inhibit HIV reactivation ex vivo. JCI Insight. 2024;9(19):e183084. doi: 10.1172/jci.insight.183084 39163135 PMC11466185

[113] Geller R, Taguwa S, Frydman J. Broad action of Hsp90 as a host chaperone required for viral replication. Biochim Biophys Acta. 2012;1823(3):698–706. doi: 10.1016/j.bbamcr.2011.11.007 22154817 PMC3339566

[114] Basha W, Kitagawa R, Uhara M, Imazu H, Uechi K, Tanaka J. Geldanamycin, a potent and specific inhibitor of Hsp90, inhibits gene expression and replication of human cytomegalovirus. Antivir Chem Chemother. 2005;16(2):135–46. doi: 10.1177/095632020501600206 15889536

[115] Acchioni C, Sandini S, Acchioni M, Sgarbanti M. Co-infections and superinfections between HIV-1 and other human viruses at the cellular level. Pathogens. 2024;13(5):349. doi: 10.3390/pathogens13050349 38787201 PMC11124504

[116] Sauter D, Hotter D, Van Driessche B, Stürzel CM, Kluge SF, Wildum S, et al. Differential regulation of NF-κB-mediated proviral and antiviral host gene expression by primate lentiviral Nef and Vpu proteins. Cell Rep. 2015;10(4):586–99. doi: 10.1016/j.celrep.2014.12.047 25620704 PMC4682570

[117] Gummuluru S, Emerman M. Cell cycle- and Vpr-mediated regulation of human immunodeficiency virus type 1 expression in primary and transformed T-cell lines. J Virol. 1999;73(7):5422–30. doi: 10.1128/JVI.73.7.5422-5430.1999 10364289 PMC112598

[118] Fassati A, Goff SP. Characterization of intracellular reverse transcription complexes of human immunodeficiency virus type 1. J Virol. 2001;75(8):3626–35. doi: 10.1128/JVI.75.8.3626-3635.2001 11264352 PMC114854

