# Supplementary figures and images for: Heat shock protein 90 is a chaperone regulator of HIV-1 latency
Source: PLoS Pathog. 2025 Apr 1;21(4):e1012524. doi: 10.1371/journal.ppat.1012524 (PMC11981193; doi:10.1371/journal.ppat.1012524)

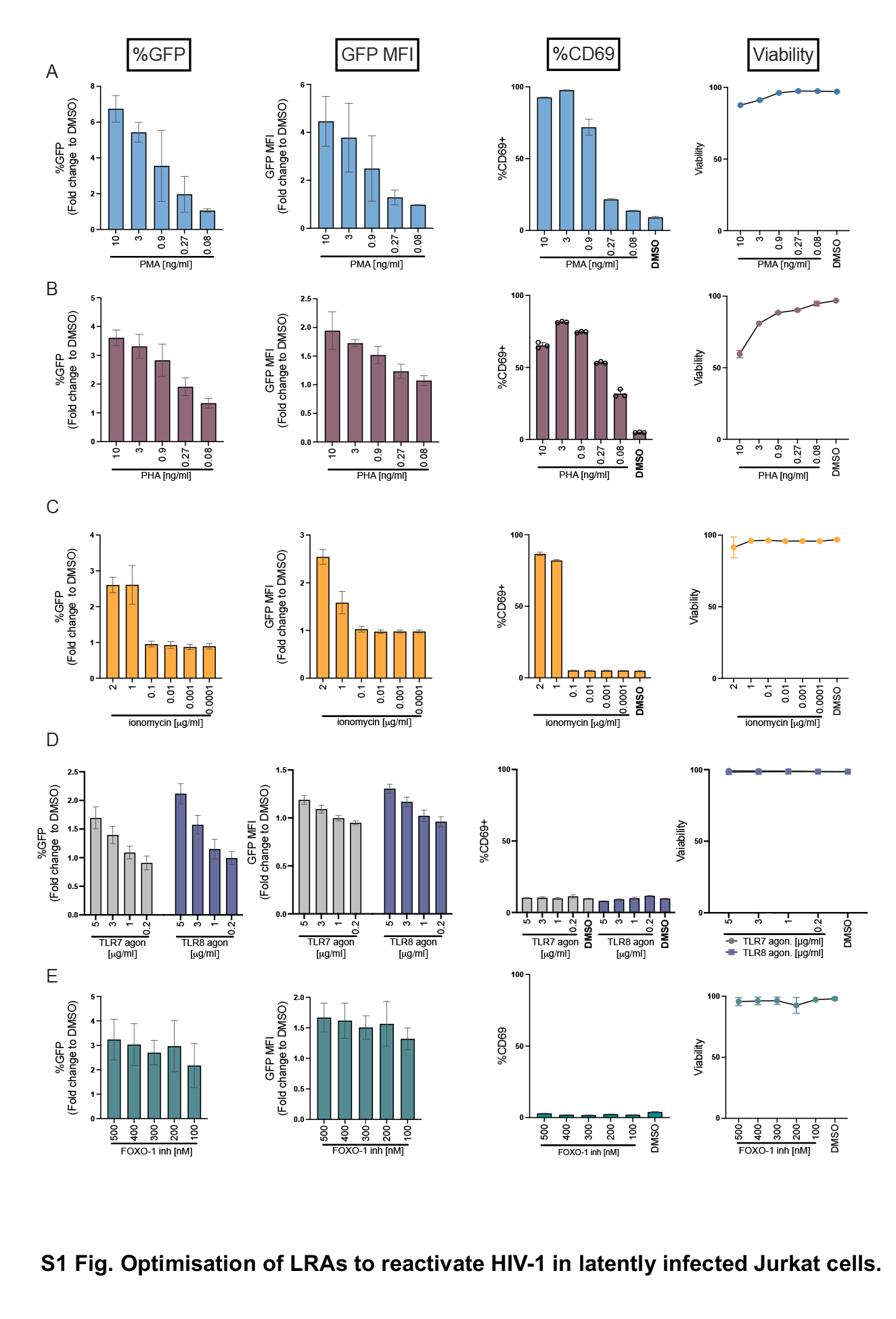

Supplement: S1 Fig — Latently infected Jurkat cells were activated for 24 hours (48 hours for the FOXO-1 inhibitor) with five different concentrations of (A) PMA, (B) PHA, (C) Ionomycin, (D) TLR7 or TLR8 agonists and (E) the FOXO-1 inhibitor. The cells were then analysed by flow cytometry to measure, from left to right, the percentage of GFP+ cells, GFP MFI, the percentage of CD69+ cells and cell viability using the same gating strategy shown in Fig 1E. Bar graphs show the average values ± SD (n = 6 for GFP) and (n = 3 for viability, CD69). Significance was calculated using a one-way ANOVA with Dunnett’s correction. *=p≤0.05; **=p≤0.01; ***=p≤0.001; ****=p<0.0001. (TIF) [file ppat.1012524.s001.tif]

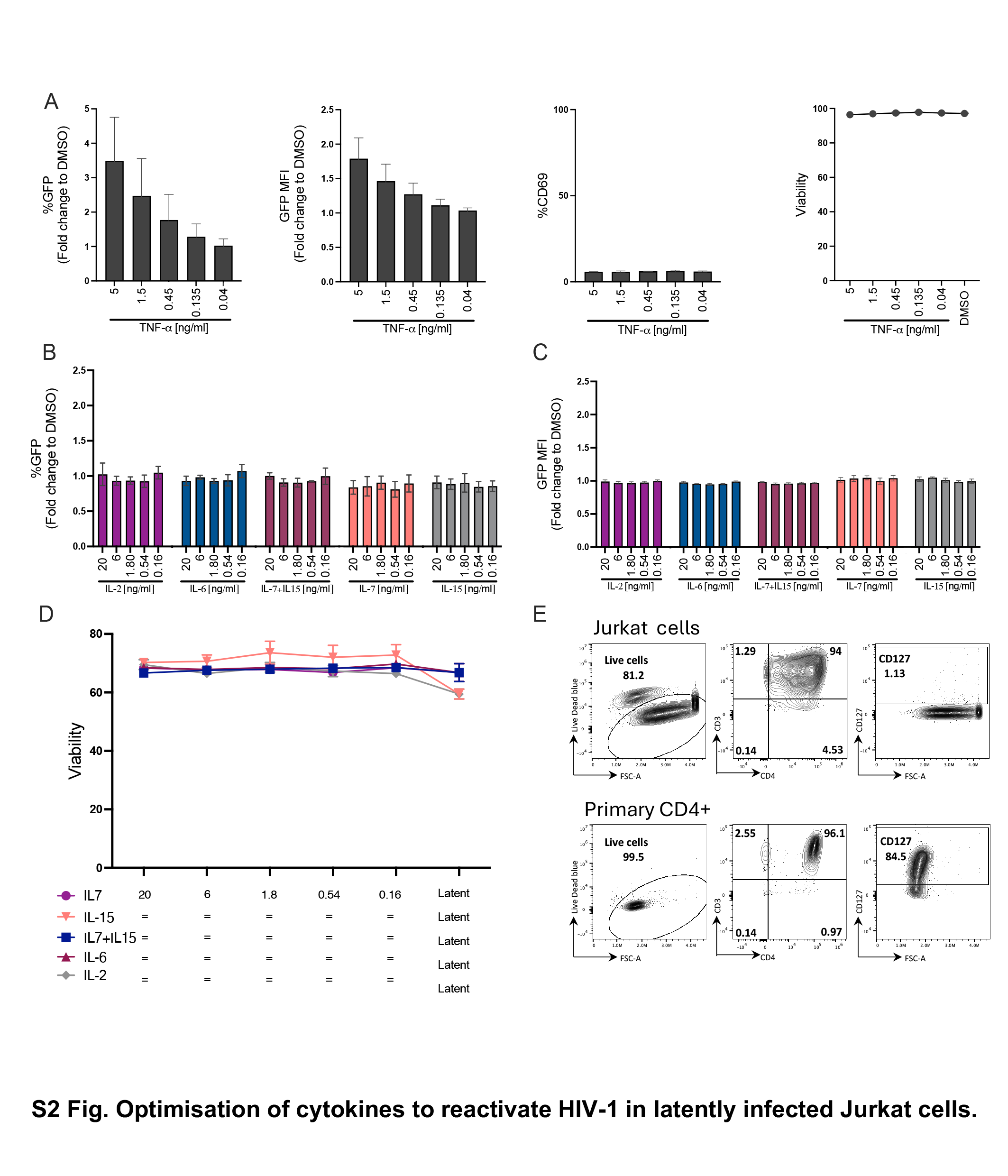

Supplement: S2 Fig — (A) Latently infected Jurkat cells were activated for 24 hours with different concentrations of TNF-α and analysed by flow cytometry to measure, from left to right, the percentage of GFP+ cells, GFP MFI, the percentage of CD69+ cells and cell viability using the same gating strategy shown in Fig 1E, n=6 for GFP and n = 3 for viability and CD69. (B) latently infected cells were stimulated with different concentrations of IL-2, IL-6, IL-7+IL-15, IL-7 alone or IL-15 alone and analysed by flow cytometry to measure the percentage of GFP+ cells and (C) the GFP MFI. Bar graphs show the average values ± SD, n = 3. (D) Cell viability was analysed by flow cytometry using forward vs. side-scatter profiles. (E) Jurkat cells and primary CD4+ T cells were stained for CD127, the IL-7 receptor. A representative flow cytometry plot shows the percentage of CD127+ cells in CD4+ T cells. (TIF) [file ppat.1012524.s002.tif]

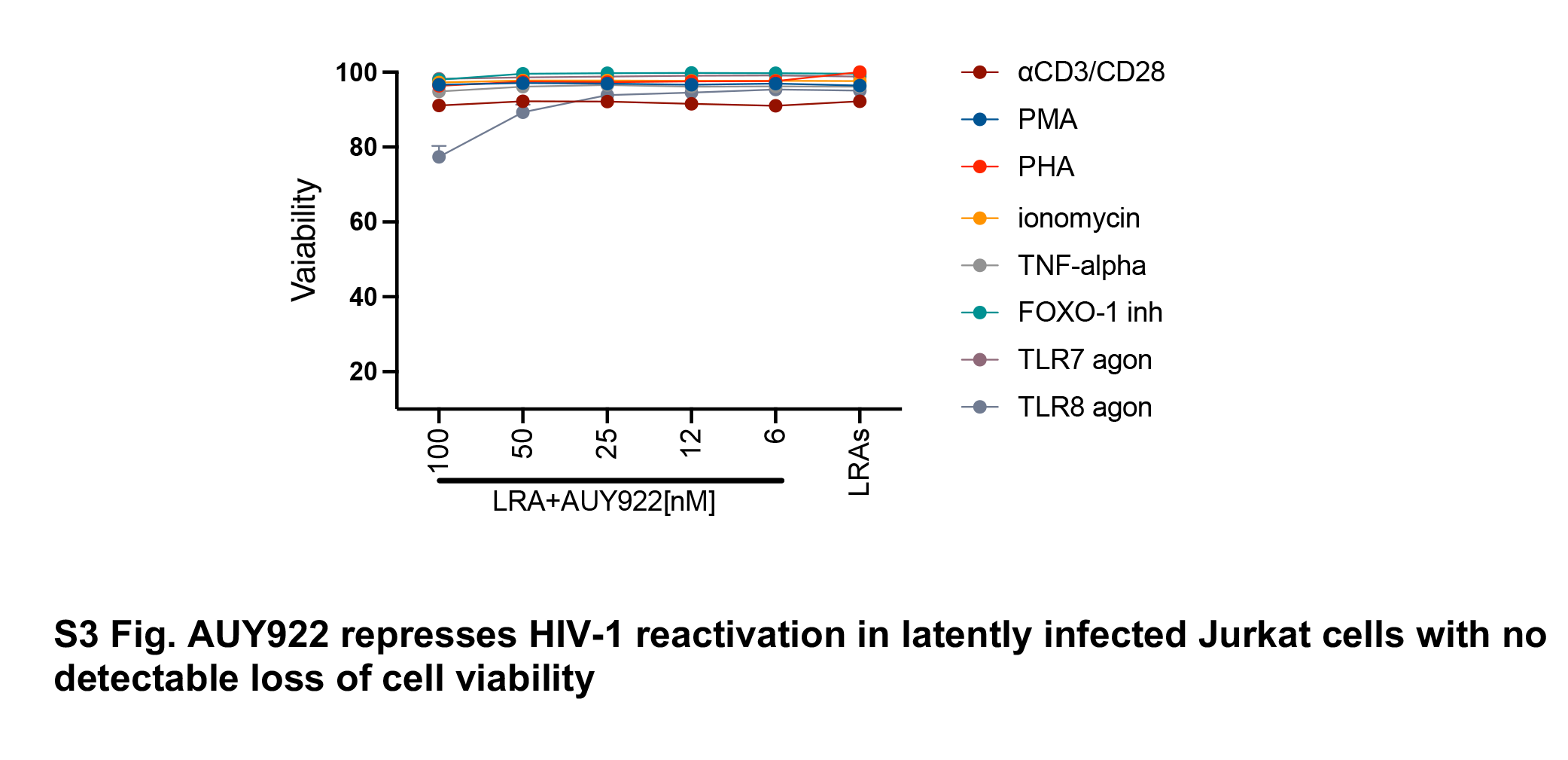

Supplement: S3 Fig — Latently infected Jurkat cells were treated with a fixed concentration of LRAs alone or with different concentrations of AUY922 for 24 hours, except for FOXO-1, which was incubated for 48 hours. Cells were then stained with live or dead blue stain and analysed by flow cytometry to assess cell viability. (TIF) [file ppat.1012524.s003.tif]

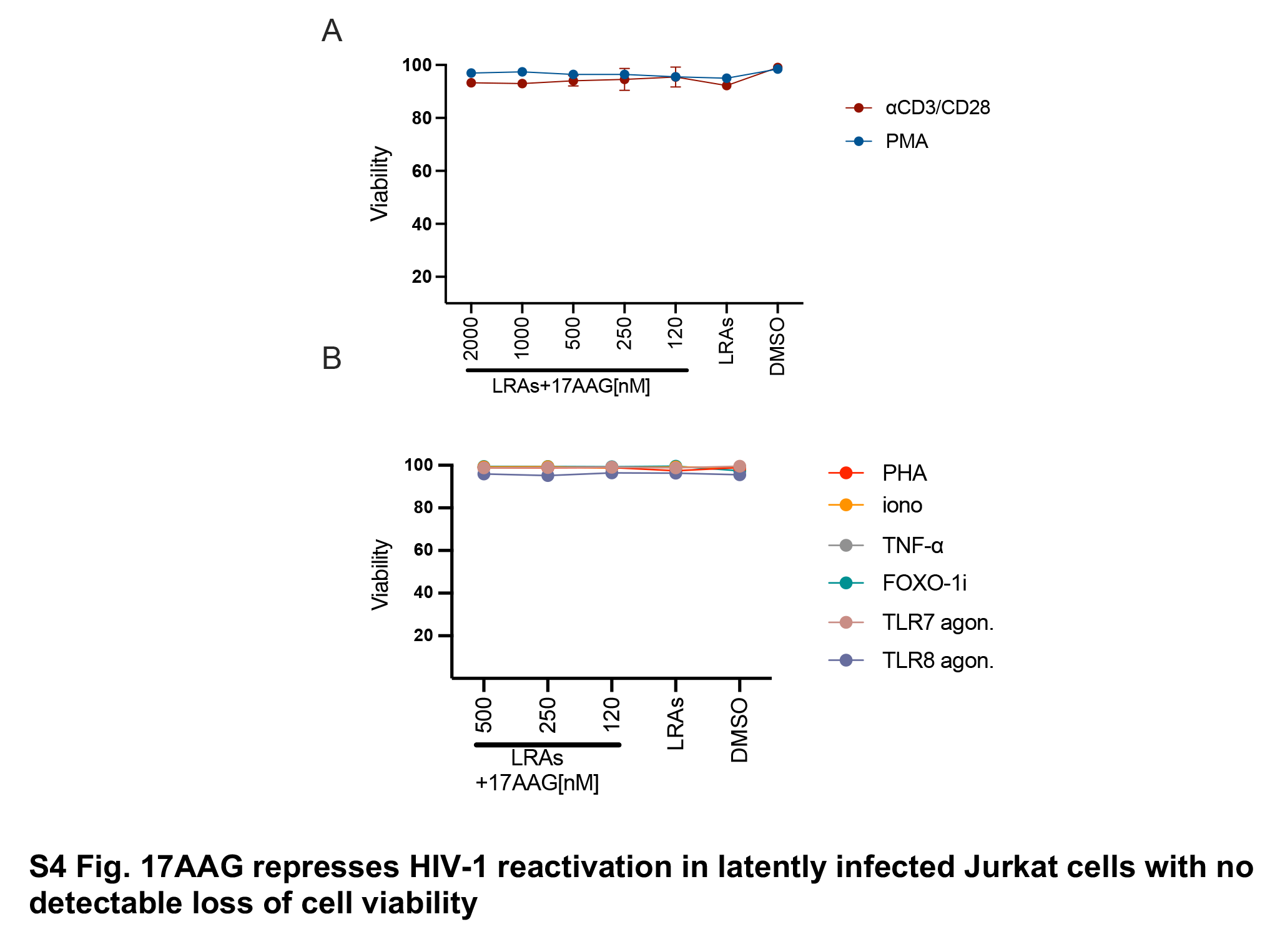

Supplement: S4 Fig — Latently Jurkat cells were treated with a fixed concentration of LRAs (anti-CD3/CD28 Abs or PMA in panel A; PHA, ionomycin, TNF-α, FOXO-1 inhibitor, TLR7 or 8 agonists in panel B) alone or with different concentrations of 17AAG for 24 hours, except for FOXO-1, which was incubated for 48 hours. Cells were then stained with live or dead blue stain and analysed by flow cytometry to assess cell toxicity. (TIF) [file ppat.1012524.s004.tif]

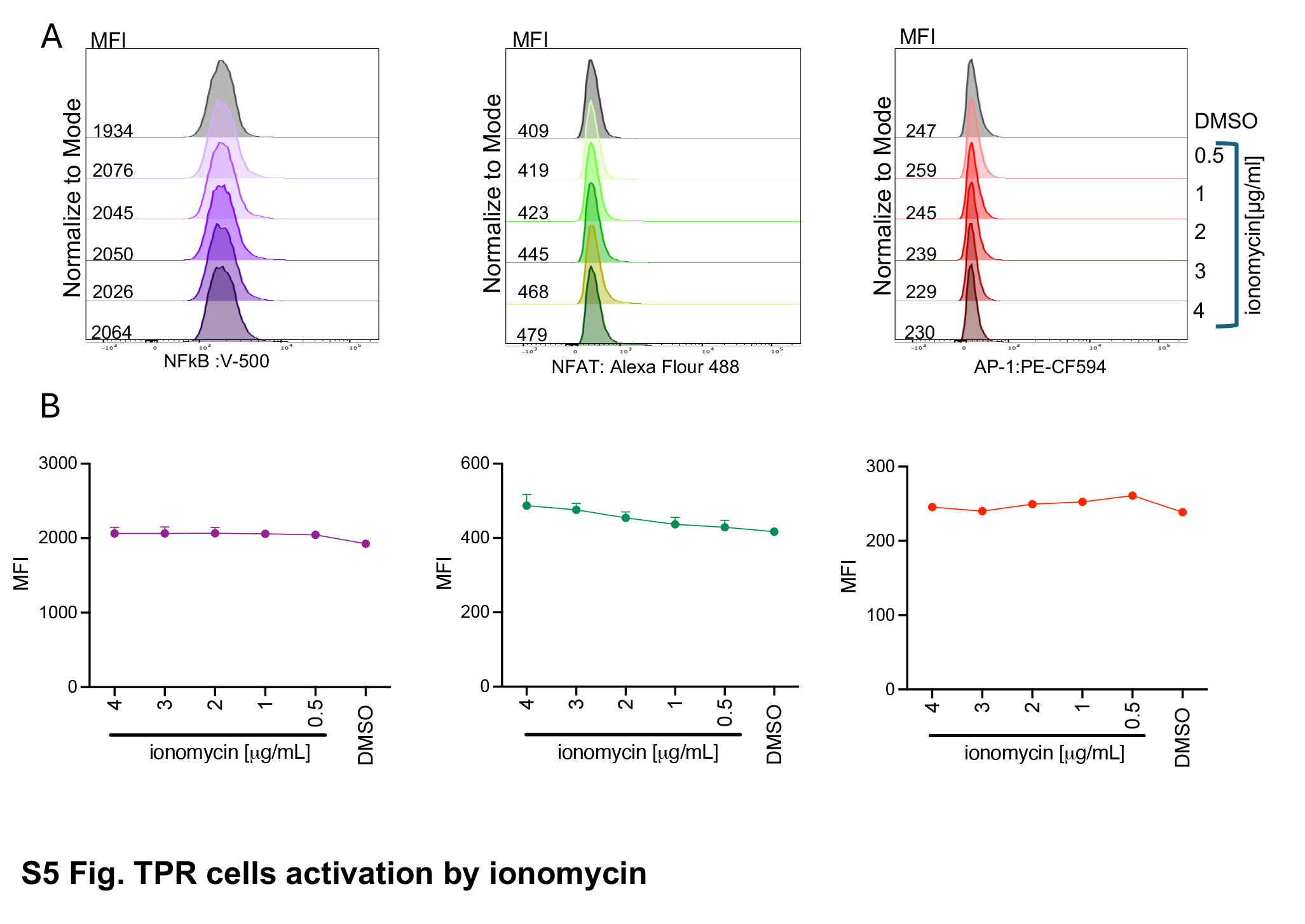

Supplement: S5 Fig — TPR cells were stimulated with different concentrations of ionomycin as indicated. (A) MFI was measured by flow cytometry to detect activation of NF-kB (left panel), NFAT (middle panel) and AP-1 (right panel). (B) Graph showing average MFI values ± SD for each transcription factor, (n= 3). (TIF) [file ppat.1012524.s005.tif]

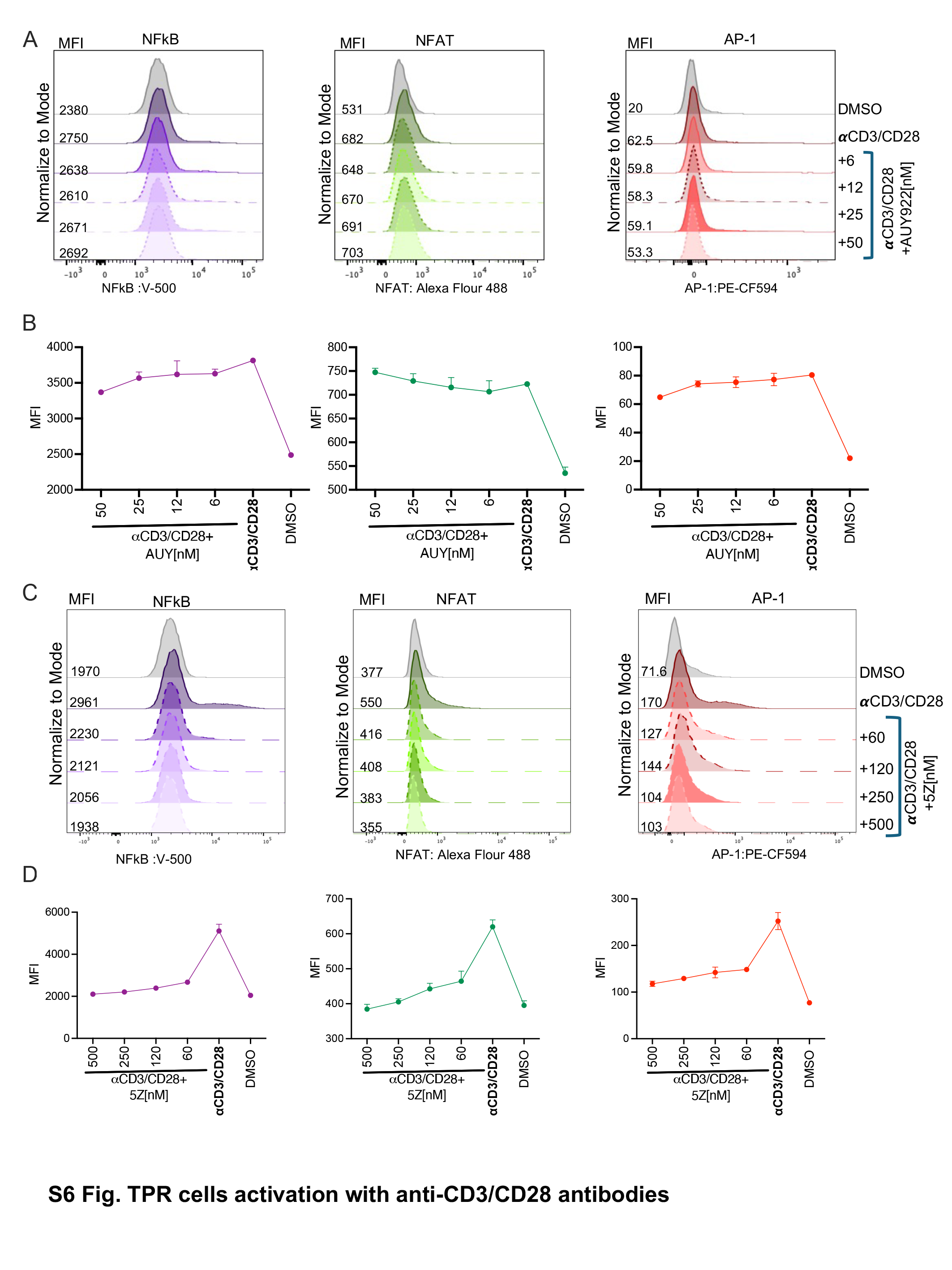

Supplement: S6 Fig — (A) TPR cells expressing a reconstituted TCR were stimulated with anti CD3 [1μg/ml]/ anti CD28 [2μg/ml] antibodies in the presence of the indicated concentrations of AUY922. MFI was measured by flow cytometry to detect activation of NF-kB (left panel), NFAT (middle panel) and AP-1 (right panel). (B) Graph showing average MFI values ± SD for each transcription factor (n= 3). (C) The same TPR cells were stimulated with anti CD3 [1μg/ml]/ anti CD28 [2μg/ml] antibodies in the presence of the indicated concentrations of 5Z. MFI was measured by flow cytometry to detect activation of NF-kB (left panel), NFAT (middle panel) and AP-1 (right panel). (D) Graph showing average MFI values ± SD for each transcription factor (n= 3). (TIF) [file ppat.1012524.s006.tif]

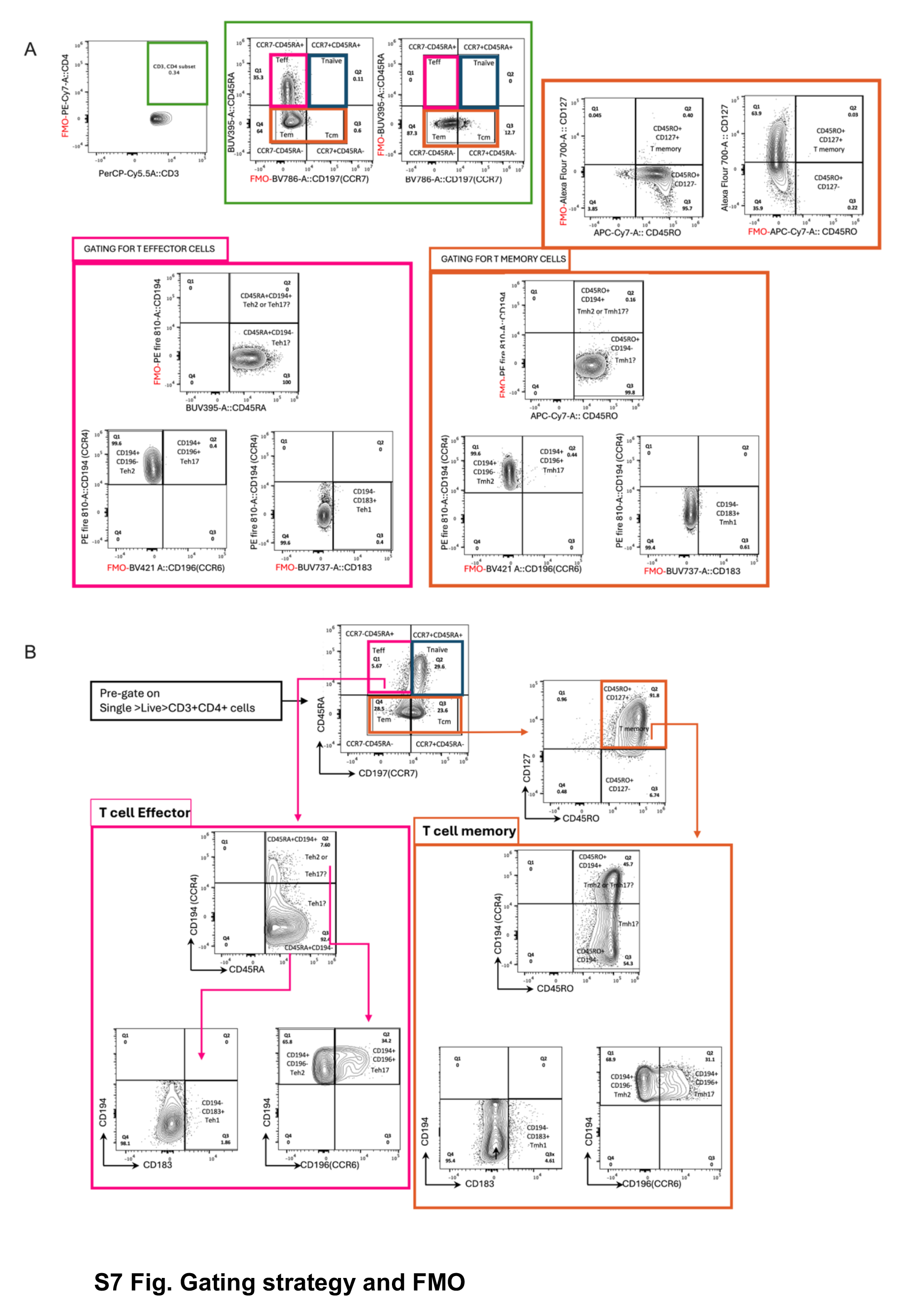

Supplement: S7 Fig — (A) Primary CD4+ T cells were analysed by spectral flow cytometry and positive gates were established by staining with the 18-antibody panel minus one (fluorescence minus one or FMO). (B) Representative flow cytometry plots and gating strategy of T cell subsets from one donor gated from live CD3+CD4+ cells. (TIF) [file ppat.1012524.s007.tif]

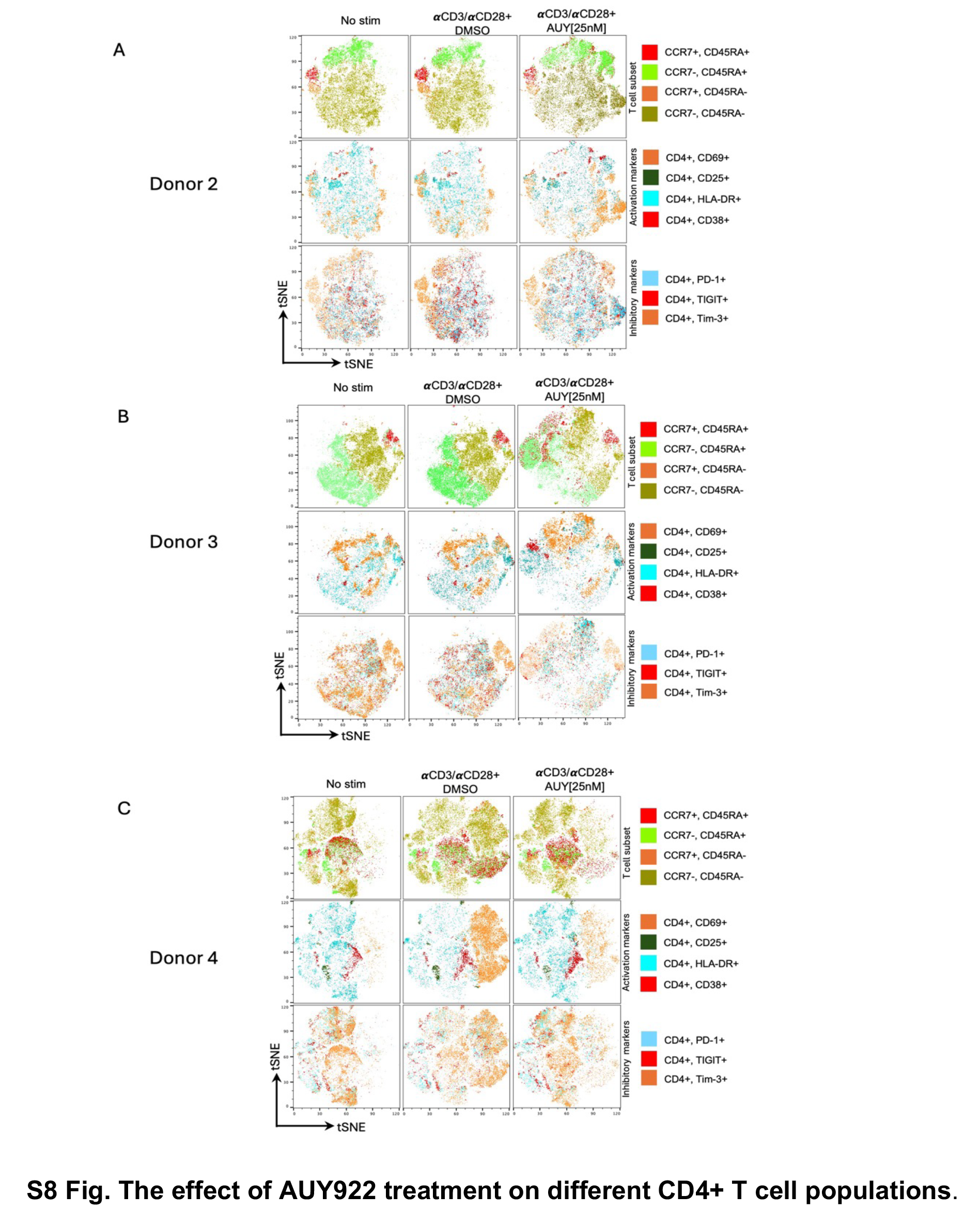

Supplement: S8 Fig — CD4+ T cells were isolated from PBMCs and treated with IL-2 only (No stim) or anti-CD3/CD28 Abs + IL-2 for 72 hours, and AUY922 [25 nM] or DMSO added 48 hours post stimulation. Cells were analysed by flow cytometry 24 hours after the addition of AUY922. tSNE data of the T subsets, activation, and inhibitory markers were generated by FlowJo. A) tSNE plot for donor 2. B) tSNE plot for donor 3. C) tSNE plot for donor 4. (TIF) [file ppat.1012524.s008.tif]

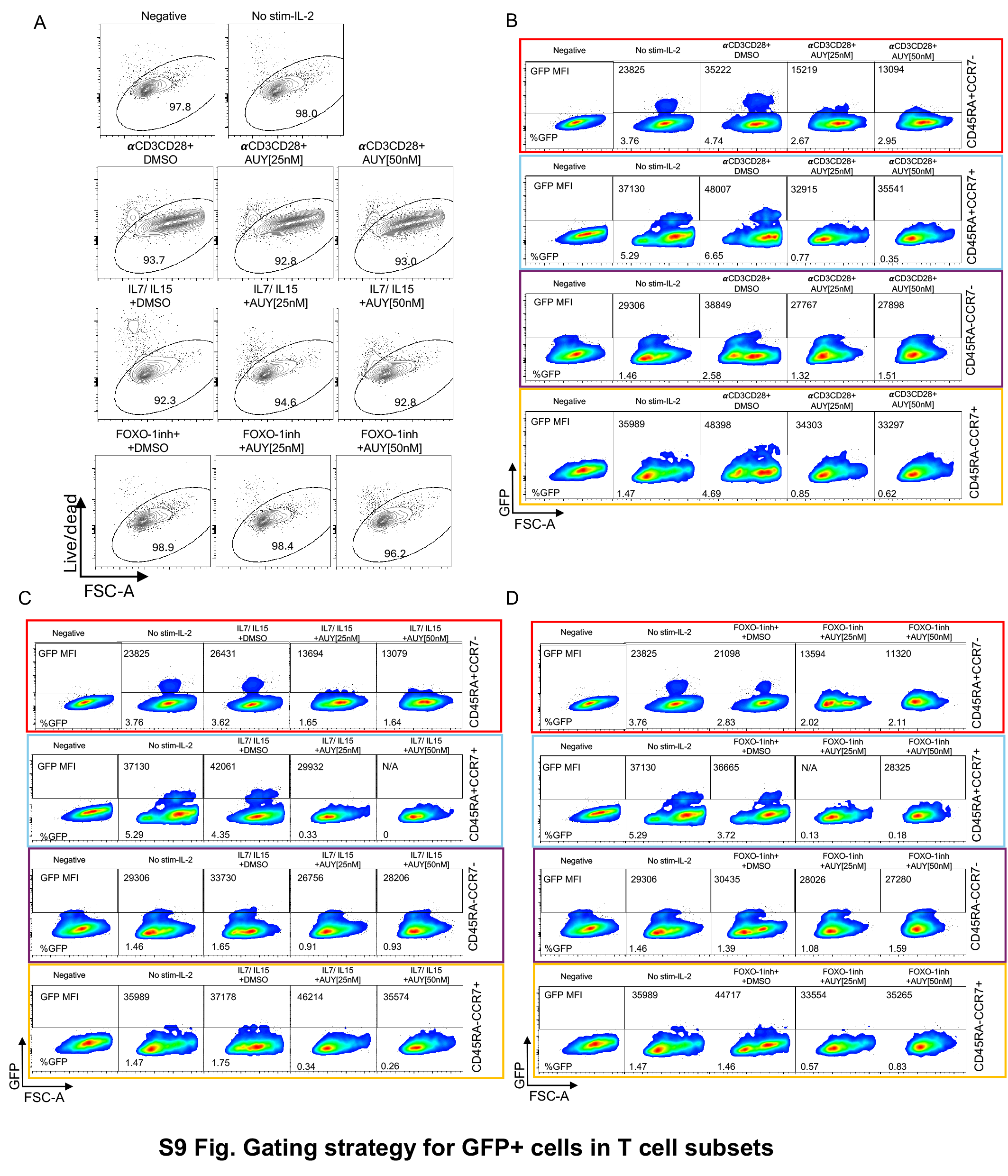

Supplement: S9 Fig — A) Representative flow cytometry plots of Live/dead gating. (B-D) Representative flow cytometry plots of GFP+ cells in T cell subsets from one donor gated from live CD3+CD4+ cells after stimulation with anti-CD3/CD28 antibodies (B), IL7/IL15 (C), or FOXO-1 inhibitor (D). (TIF) [file ppat.1012524.s009.tif]

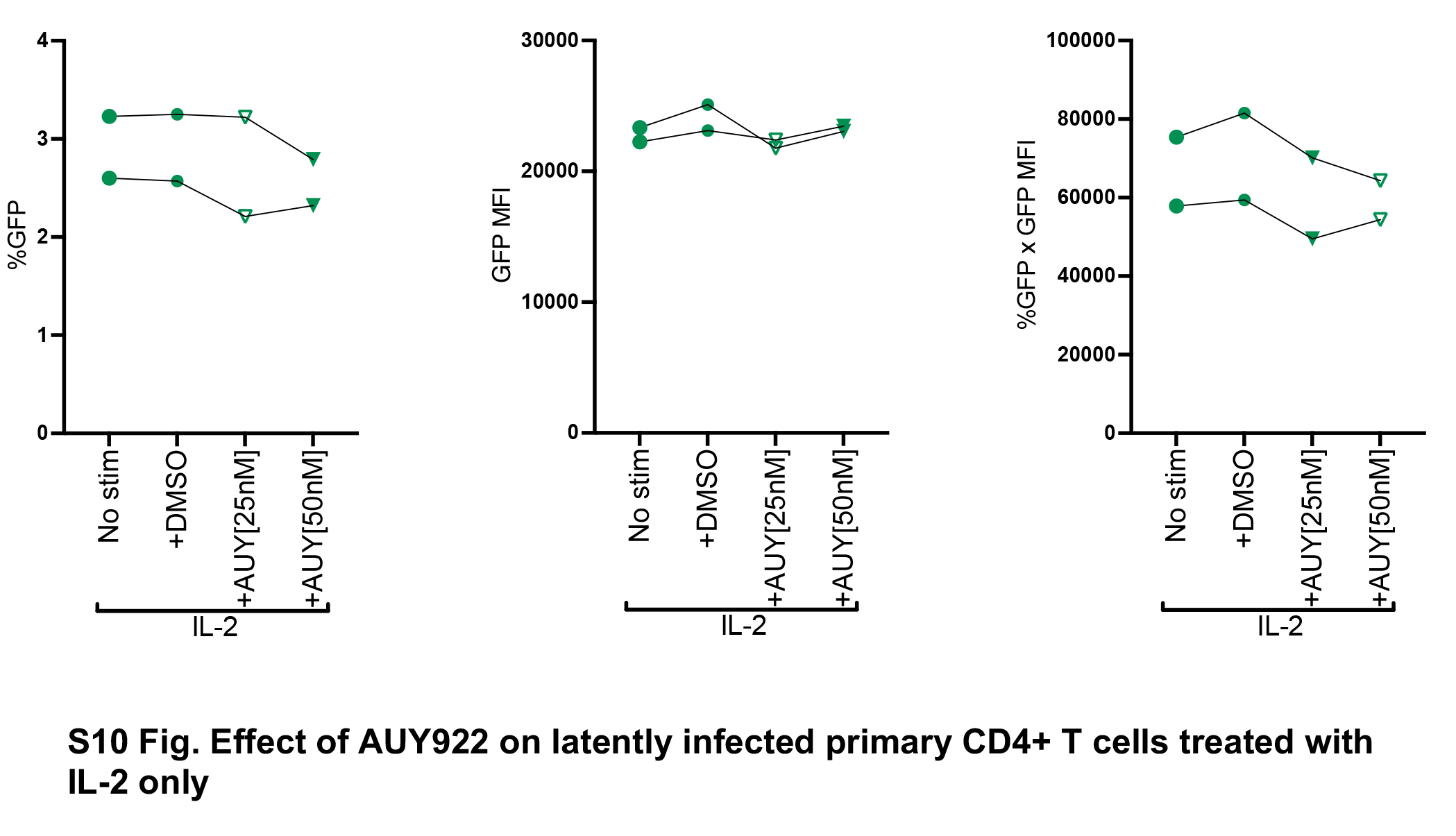

Supplement: S10 Fig — Latently infected primary CD4+ T cells were generated ex vivo as described in Fig 7A. On day 9 or 11, cells were treated with AUY922 [25 nM or 50 nM] or DMSO in the presence of IL-2. Graphs showing the results for 2 donors: GFP MFI (left panel), percentage of GFP+ cells (middle panel), and combined % GFP x MFI in the CD45RA- CD45RO+ population. (TIF) [file ppat.1012524.s010.tif]

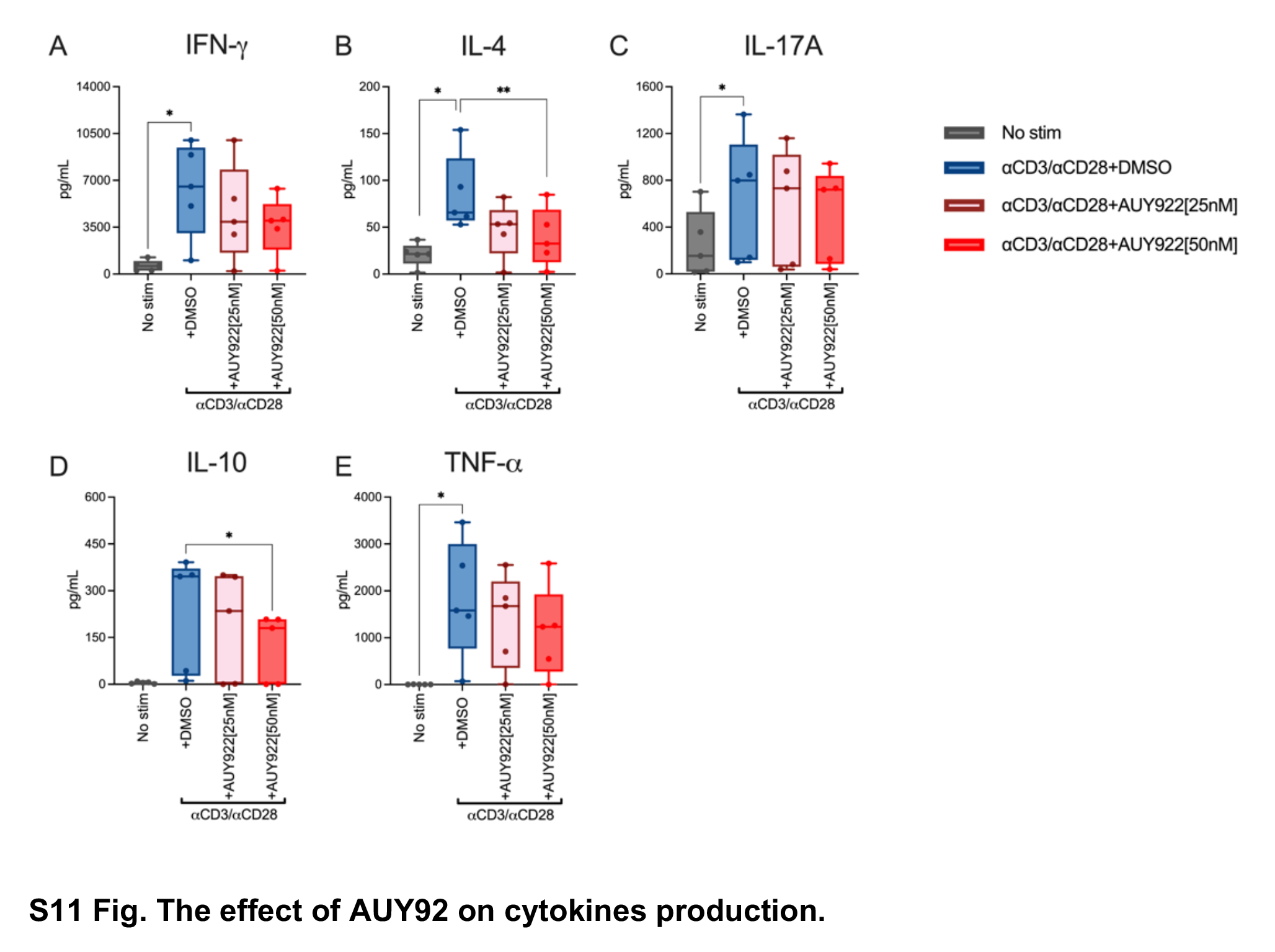

Supplement: S11 Fig — Supernatant from latently infected primary CD4+ T cells, which were re-stimulated with anti-CD3/CD28 Abs in the presence or absence of DMSO, AUY922 (25 nM), or AUY922 (50 nM) was collected 48 hours after re-stimulation and used to measure different cytokines concentration. (A) IFN-γ, (B) IL-4, (C) IL-17A, (D) IL-10, and (E) TNF-α. Data are presented as mean ± standard error of the mean (SEM) n=5. Concentrations are shown in pg/ml. Statistical significance was determined using a Paired two-tailed Student’s t-test. *P < 0.05, **P < 0.01, ***P < 0.001. Pairwise comparisons were: No stim-IL-2 and anti-CD3/CD28; anti-CD3/CD28 and AUY922 25nM; anti-CD3/CD28 and AUY922 50 nM. (TIF) [file ppat.1012524.s011.tif]
